# Neural signatures of risk-taking adaptions across health, bipolar disorder, and lithium treatment

**DOI:** 10.1038/s41380-025-02900-w

**Published:** 2025-01-29

**Authors:** Jacqueline Scholl, Priyanka Panchal, Natalie Nelissen, Lauren Z. Atkinson, Nils Kolling, Kate EA Saunders, John Geddes, Matthew FS Rushworth, Anna C. Nobre, Paul J. Harrison, Catherine J. Harmer

**Affiliations:** 1 Université Claude Bernard Lyon 1, CNRS, Inserm, Lyon Neuroscience Research Centre U1028 UMR 5292, PsyR2 team, Centre Hospitalier Le Vinatier, Bron, France; 2 Oxford Centre for Human Brain Activity (OHBA), Wellcome Centre for Integrative Neuroimaging (WIN), Department of Psychiatry, University of Oxford, Oxford, UK; 3 Department of Psychiatry, University of Oxford, Oxford, UK; 4 DWP Digital, Leeds, UK; 5 Université Claude Bernard Lyon 1, Inserm, Stem Cell and Brain Research Institute U1208, Bron, France; 6 Oxford Health NHS Foundation Trust, Warneford Hospital, Oxford, UK; 7 Department of Experimental Psychology, Wellcome Centre for Integrative Neuroimaging (WIN), University of Oxford, Oxford, UK; 8 Department of Psychology and Wu Tsai Institute, Yale University, New Haven, CT USA

**Keywords:** Neuroscience, Diagnostic markers

## Abstract

Cognitive and neural mechanisms underlying bipolar disorder (BD) and its treatment are still poorly understood. Here we examined the role of adaptations in risk-taking using a reward-guided decision-making task. We recruited volunteers with high (*n* = 40) scores on the Mood Disorder Questionnaire, MDQ, suspected of high risk for bipolar disorder and those with low-risk scores (*n* = 37). We also recruited patients diagnosed with BD who were assigned (randomized, double-blind) to six weeks of lithium (*n* = 19) or placebo (*n* = 16) after a two-week baseline period (*n* = 22 for FMRI). Participants completed mood ratings daily over 50 (MDQ study) or 42 (BD study) days, as well as a risky decision-making task and functional magnetic resonance imaging. The task measured adaptation of risk taking to past outcomes (increased risk aversion after a previous win vs. loss, ‘outcome history’). While the low MDQ group was risk averse after a win, this was less evident in the high MDQ group and least so in the patients with BD. During fMRI, ‘outcome history’ was linked to medial frontal pole activation at the time of the decision and this activation was reduced in the high risk MDQ vs. the low risk MDQ group. While lithium did not reverse the pattern of BD in the task, nor changed clinical symptoms of mania or depression, it changed reward processing in the dorsolateral prefrontal cortex. Participants’ modulation of risk-taking in response to reward outcomes was reduced as a function of risk for BD and diagnosed BD. These results provide a model for how reward may prime escalation of risk-related behaviours in bipolar disorder and how mood stabilising treatments may work.

## Introduction

Bipolar disorder (BD) is typically characterized by episodes of depression or mania, lasting weeks and months. Lithium is the most effective mood stabiliser for management of BD, reducing the frequency of both manic and depressive episodes [1]. While fluctuating mood episodes have traditionally be seen as lasting weeks or months, more recent work has shown that, in fact, patients with BD show large day-to-day fluctuations in mood even when symptoms are in the non-clinical range [2] and that this is affected by lithium treatment [3]. Understanding the processes underpinning bipolar disorder may help us develop and assess more effective treatment approaches.

From a computational psychiatry perspective, two causes for mood fluctuations in bipolar disorder could be considered. First, mood fluctuations could be the result of either increased and prolonged responses to valanced outcomes. Recent work from the field of reinforcement learning has suggested that destabilizing positive feedback cycles between mood and perceptions of rewards may contribute to BD [4–7]: In people with subclinical symptoms of BD, positive or negative surprises were found to affect the neural and behavioural responses to reward and punishments. In particular, symptoms were associated with an increase in reward value after a positive surprise. This kind of reward sensitivity has been linked to later changes in mood, suggesting a route by which escalation of reward responses may translate into clinical symptoms [4]. Second, mood fluctuations could be the result of reduced behaviours that stabilize mood. Using momentary ecological monitoring has revealed that in the healthy state, when mood fluctuates, people self-report using strategies to re-establish mood homeostasis such as engaging with aversive activities when they are in a good mood [8]. This strategy is reduced in people with depression or low mood [9]. However, it is yet unclear whether regulating behaviour is also reduced in BD. In the lab, adaptations of behaviour to past outcomes have been studied in the field of decision-making, revealing temporal interdependencies. For example, people show ‘biases’ such as ‘loss chasing’ [10] (taking more risks to try and recover losses). Here, we used a lab-based task that allowed us to test the impact of BD and its’ treatment on both putative processes.

Optimal decision making involves interplay between frontostriatal systems, which play a role in motivation, reward value and its regulation. The ventral striatum and the ventromedial prefrontal cortex (vmPFC) are implicated in reward anticipation as well as its hedonic impact [11, 12]. vmPFC is further implicated in the evaluation of options [13], including tracking of past reward outcomes [14]. We would therefore expect that if bipolar disorder affects the adaptation of behaviour to past outcomes, these signals in the vmPFC should be changed. By contrast, activity in the dorsolateral PFC is associated with regulation of behaviour towards reward, including self-regulation of reward craving [15, 16]. Previous work has linked bipolar disorder to increased reward related striatal signalling, coupled with altered patterns of ventromedial and dorsolateral PFC engagement [17] and interaction [18], while a meta-analysis [19] has highlighted a role for orbitofrontal cortex abutting dlPFC.

Here, we have built on these findings to test whether a gradient across a bipolar disorder spectrum (i.e. from low risk to diagnosed bipolar disorder), was linked to changed behavioural adaptation (risk taking) from trial to trial in response to reward/loss outcomes. For this, we recruited 40 volunteers with high scores on the mood disorder questionnaire (MDQ [20]), at suspected high risk for bipolar disorder, 37 volunteers with low scores, and 35 treatment seeking patients with diagnosed BD (*n* = 22 for FMRI). To assess whether behaviour and naturally occurring daily-life mood fluctuations were related, participants completed up to 50 longitudinal testing sessions at home. To understand the neural mechanisms of risk adaptation behaviour, we measured brain activity with fMRI. To test the causal effect of a commonly prescribed mood-stabilizing drug, lithium, 19 patients were randomly assigned to receive six weeks of lithium treatment (dose titrated individually to plasma levels of 0.6–1 mmol/L) and 16 to placebo treatment in a double-blind design.

We hypothesized that BD and risk for BD would be associated with reduced adaption of risk taking behaviour (i.e. choice being less connected to previous experience of a win or a loss), which would be associated with changes in vmPFC and dlPFC signalling of previous win/loss experiences during fMRI. We also hypothesized that these behavioural and neuroimaging differences would be normalised following six weeks of lithium vs placebo treatment in BD.

## Methods

### Participants and ethics statement

Participants were recruited in two separate studies (see below). The non-interventional study was approved by the local ethics committee (MSD-IDREC-C2-2014-023) and the interventional study by the National Research Ethics Service Committee South Central – Oxford A (15/SC/0109) and the Oxford Health NHS Foundation Trust. Participants gave informed consent and were reimbursed for taking part in the study. All methods were performed in accordance with the relevant guidelines and regulations.

#### Volunteers at suspected high vs low risk of bipolar disorder

Participants were recruited through local advertisement and from pools of previous participants. In an online pre-screening session, participants completed the Mood Disorders Questionnaire (MDQ [20]), a self-report screening instrument to identify risk for bipolar disorder. Participants were only invited for a full screening session if they scored either <5 points (‘low MDQ’ group, *n* = 37 included, at presumed low risk for bipolar disorder); or ≥7 (‘high MDQ’ group, *n* = 40 included). The screening verified that several of these symptoms measured with the MDQ happened during the same period of time. Structured clinical interviews with the SCID revealed that 5 of this group met criteria for bipolar disorder, despite not having received a formal diagnosis or seeking treatment. See Supplementary Method [1A] for detailed exclusion criteria.

#### Patients with BD

Participants were recruited through the BD Research Clinic (Oxford). All participants met criteria for BD-I (*n* = 7), BD-II (*n* = 27) or BD not otherwise specified (BD-NOS, *n* = 1), based on structured clinical interview. All participants were outside major mood episodes requiring immediate treatment. Full exclusion criteria are provided in the Supplementary Materials [1B]. Participants were assigned to placebo (*n* = 16) or lithium (*n* = 19), in a randomised double-blind design, see below.

### Study design

#### Volunteers

We measured participants’ mood and behaviour in a cognitive task longitudinally five times a week over ten weeks. Brain activity during the same task was measured during an MRI scan. The data here were part of a larger study (Supplementary Method [1B]).

#### Patients with BD

This study was a randomised, 6-week, double-blind, placebo-controlled trial [21]. See Supplementary Method [1B] for full information. All participants underwent a two-week pre-randomization phase (‘baseline’) during which they completed the cognitive task and mood ratings daily at home. Due to logistic challenges, for some participants this phase lasted longer than two weeks. For the next phase (6 weeks), participants were pseudo-randomly assigned to receive either lithium (starting dose of 400 mg and then titrated to plasma levels of 0.6–1 mmol/L) or placebo in a double-blind design. Only 22 participants were fMRI compatible. Participants were invited to complete online weekly assessments of depression symptoms with the Quick Inventory of Depressive Symptomatology (QIDS, [22]) and symptoms of Mania with the Altman Self Rating Mania Scale [23].

Throughout, we performed two types of group comparisons. First, we compared across risk of BD (i.e. group as ordered factor [24] in regressions, Low MDQ ≤ High MDQ ≤ patients with BD), subsequently referred to as ‘bipolar disorder gradient’. Ordered factors in regression imply a relationship of order between the groups, this does not have to be a linear relationship (i.e. the difference low MDQ to high MDQ can be larger or smaller [but of same sign] than the difference high MDQ to patients with BD). MDQ was not measured in the patient group. As this involved data from the BD group before assignment to lithium or placebo, all participants were included. Significant results were post-hoc followed up comparisons of the individual groups (t-tests). Second, we tested for the effects of lithium treatment as drug (lithium/placebo) x time point (pre, i.e. baseline/post) interactions.

### ‘Wheel of fortune’ task

#### Trial structure

**Fig. 1:**
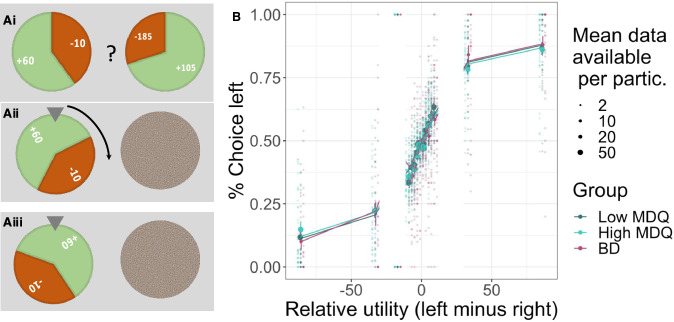
Task design and longitudinal behaviour. **A** On each trial, participants chose between two gambles (‘wheel of fortune’) that differed in their probability of winning or losing points and in the number of points that could be won or lost. Once participants had chosen an option, the alternative was hidden, and the chosen wheel started spinning until finally landing on the win or loss. **B** Participants’ choices (left vs. right option) were guided by the relative utilities (reward utility – i.e., probability * magnitude – minus loss utility): the higher the utility of the left option, the more it was chosen. The computational model (lines) captured behaviour (dots with error bars) well. Data were combined across all testing sessions (up to 50) per participant (20 trials per session). Error bars show the standard error of the mean, and the size of the dots indicates the number of data points available.

On each trial of the task, participants were given two options shown side-by-side. In the at-home version, these were wheels of fortune (Fig. 1A). In the fMRI version, they were instead presented as bars. Each option had three attributes: probability of winning vs. losing (size of green vs. red area), magnitude of possible gain (number on green area, 10–200), and magnitude of possible loss (number on red area, also 10–200). After participants chose one option, the wheel of fortune started spinning and then randomly landed on either win or loss. Finally, participants were shown their updated total score. The experiment was designed so that most choices were difficult, i.e., the options were very similar in expected value, i.e. relative utility (reward magnitude * probability; 90% of choices were not more than 20 points apart; 76% not more than 5 points apart, Fig. 1B, Supplementary Fig. S1).

#### Timings and number of trials

Each day, participants rated their positive and negative mood using the Positive and Negative Affect Schedule – Short Form, PANAS-SF [25]. They also gave an overall rating of their mood (‘How are you feeling’, referred to here as ‘happiness VAS’) using a slider ranging from ‘very unhappy’ (red sad face drawing) to ‘very happy’ (green smiley face). They then played 20 trials of the task. After the task, they repeated the happiness VAS.

In the fMRI scanner, participants played 100 trials. All timings were jittered. From the onset of options until participants could make a choice: 1–2 s; delay between participants’ response and outcomes shown: 2.7 to 7.7 s; duration of outcome shown: 1–3 s; duration of total score shown: 1–9 s; ITI: 1–9 s.

##### Behaviour

Behavioural data were analysed in R [26] (version 4.0.2) and Matlab. R-packages: Stan [27], BRMS [28, 29], dplyr [30], ggpubr [31], sjPlot [32], compareGroups [33], emmeans [34], ggsci [35].

### Group comparisons

To compare groups, instead of a standard ANOVA procedure which tests for any differences between groups, we tested for a systematic effect, i.e. bipolar disorder gradient (group as ordered factor [24], Low MDQ ≤ High MDQ ≤ patients with BD) in linear regressions, also controlling for age and gender. Models used the BRMS toolbox interface for Stan (Supplementary Methods 2). For this and all subsequent analyses, we used Bayesian Credible Intervals [36] to establish significance by the 95% CI not including zero.

### Computational models

#### Decision making

We used a computational model to capture participants’ choices. The model first computed the overall expected value (‘utility’) of each option, then made a choice (left or right option) depending on which option had the better utility, but also allowing for some random choice behaviour [37, 38].

First, the model compared the options’ utilities as displayed at the time of choice on the current trial, i.e., probability (prob) x magnitude (mag). We allowed for individual differences in sensitivity to the loss vs. reward utility (λ). We also included in the model a measure of adaptions of risk taking (i.e. loss vs win sensitivity) to past outcomes (‘outcome history’). Specifically, a parameter (γ) changed the weighting of the loss utility on the current trial depending on whether the previous trial’s outcome was a win or a loss (i.e., γ > 0 means increased sensitivity to losses after a win on the previous trial).

$${{Utility}}_{{left}}={Prob}* {{Mag}}_{{rew}}-\left({{{\rm{\lambda }}}}+{{{\rm{\gamma }}}}* {{PrevOutc}}_{\frac{{win}}{{loss}}}\right)* \left(1-{Prob}\right)* {{Mag}}_{{loss}}$$

To decide which option to choose, the model compared the utilities of the left and right options taking into account each participant’s ‘randomness’ (inverse temperature (*β*), higher numbers indicating higher choice consistency):

$$p\left({{Choice}}_{{left}}\right)=\frac{1}{1+{e}^{{{{\rm{\beta }}}}* ({{Utility}}_{{right}}-{{Utility}}_{{left}})}}$$

To allow fitting of individual sessions (20 trials), a Bayesian approach was implemented that allowed specifying priors for each parameter (Supplementary Methods [2C]). The model was validated using simulations and model comparisons (Supplementary Tables S1–3 and Supplementary Methods [2A–B]).

### Group differences

To assess group differences, we entered the session-wise parameters into hierarchical regressions (using BRMS). This allowed us to take into account that parameters might change over days of testing, as well as individual differences in the means and variability (standard deviation) across sessions. For example:

Mean: invTemp(β) ~ 1 + day + group + Age + Gender + (1 + day | ID),

And error term: sigma ~ 1 + group + Age + Gender + (1|ID))

The effect of lithium (vs. placebo) was tested analogously:

Mean: invTemp(β) ~ 1 + day + group*pre, i.e. baseline/post + Age +Gender + number_days_baseline + (1 + day | ID)

These models were used for group comparisons of mean parameters (Supplementary Methods 2C, D). Variabilities of parameters over days were not compared as model validation (Supplementary Table S1) suggested poor recovery. Mood data (positive and negative PANAS, happiness VAS) were analysed using similar regressions (Supplementary Methods 2D) to assess group differences in mood (mean or variability) or the relationship between task outcomes and changes in happiness VAS.

### Model-free analyses of behaviour

To test that participants could perform the task, i.e., that their choices were sensitive to expected value, we binned their choices (% left vs. right option) according to the overall utility difference between the two options (i.e., left vs. right reward utility minus loss utility, utility = probability*magnitude).

To test sensitivity to risk of losses, as has been previously reported to be affected in BD [39, 40], we refined the binning of choices (as above) by further splitting the data according to win and loss utility (i.e. probability * magnitude).

We next analysed behaviour for adaptions of risk taking to past outcomes by considering how participants change their behaviour – here risk-taking (avoidance of potential losses) – based on win/loss outcomes on previous trials (‘outcome history’ effect). For this, we computed how their choices differed after a win or loss on the previous trial (difference % choosing option with lower potential loss [loss utility] after win minus after a loss). We focused on the most extreme (lowest/highest) loss utility bins from the analyses above (‘taking loss utility more into account’) as adaptation to past trial outcomes by taking loss more into account (i.e. a multiplicative effect) should most strongly affect choices the more dissimilar the loss utilities of the two options.

### MRI acquisition

Data from all 77 high and low MDQ volunteers and 13 patients with BD were collected on a 3T Siemens Magneton Trio. Data from 9 patients with BD were collected at a different site using a Siemens Magneton PRISMA. Group comparisons include scanner as a control regressor. Scan protocols were carried out following [14], Supplementary Methods [3A].

### FMRI analysis – whole-brain

#### General approach

Data were pre-processed using FSL ([41], Supplementary Methods [3B]). Statistical analysis was performed at two levels, event-related GLM for each participant, followed by group-level mixed-effect model using FSL’s FLAME 1 [42, 43] with outlier de-weighting. Whole-brain images are all cluster-corrected (*p* < 0.05 two-tailed, FWE), voxel inclusion threshold: z < 2.3.

#### Regression designs

At the time of the decision, we looked for neural activity correlating with the utility (reward, loss) of the choice. At the time of the outcome of the gamble, we looked for neural activity related to the processing of the outcome (win/loss as continuous regressor). Decision and outcome-related activity could be dissociated due to jitter used in the experimental timing [14]. As a key measure of interest, we looked at whether there was a history effect at the time of the choice (i.e., previous trial’s gamble win/loss outcome [14, 44], analogous to the behavioural analyses). Full design information: Supplementary Methods [3C], Supplementary Fig. S2.

#### Group-level comparisons

We compared the low vs high MDQ groups (*n* = 77) in whole-brain analyses. As only 22 patients with BD were available, these group comparisons were first performed in regions of interest (ROIs) derived from comparisons of the high/low MDQ groups. As exploratory analyses, BD groups were also compared at the whole-brain level.

#### ROI analyses

Mean brain activations (COPES, contrasts of parameter estimates) were extracted for each participant. These were used to illustrate group differences and also to perform independent statistical tests (e.g., ROIs of clusters defined based on group differences of high vs. low MDQ could be used to test group differences between lithium and placebo). For this, non-hierarchical Bayesian regressions were used, also controlling for age and gender. Brain activations for the outcome history effect were correlated with the corresponding behavioural measures. For this, effects of age, gender and group (and for the patients with BD: number of days in the baseline phase pre-randomization, i.e. before the MRI scan) were first removed using regressions from both neural and behavioural measures.

## Results

We recruited four groups of participants in two separate studies (Table 1). In the group of patients with BD, based on self-report scores (Altman self-report scale, quick inventory of depressive symptomatology), in the phase before the assignment to placebo or lithium, 30% scored in the mania range and 53% scored at least moderate symptoms of depression (Table 1). Similar numbers persisted throughout treatment with lithium vs. placebo (Table 1, Supplementary Fig. S3). Participants with BD took several medications at study inclusion (Table 2).

**Table 1:** Participant demographics.

	Low MDQ	High MDQ	Bipolar lith	Bipolar pla	ANOVA all 4 groups (*p*-value)	Low vs high MDQ (*p*-value)	BD lith vs. pla (*p*-value)
#Participants	*N* = 37	*N* = 40	*N* = 19	*N* = 16			
Age	25.0 (6.61)	25.0 (7.06)	28.8 (9.81)	35.1 (13.8)	<0.001	0.974	0.137
Gender:					0.819	0.998	1
F	24 (64.9%)	27 (67.5%)	11 (57.9%)	9 (56.2%)			
M	13 (35.1%)	13 (32.5%)	8 (42.1%)	7 (43.8%)			
Diagnosis:					<0.001	0.119	0.527
BDI	0 (0.00%)	0 (0.00%)	3 (15.8%)	4 (25.0%)			
BDII	0 (0.00%)	3 (7.50%)	16 (84.2%)	11 (68.8%)			
BD NOS	0 (0.00%)	2 (5.00%)	0 (0.00%)	1 (6.25%)			
None	37 (100%)	35 (87.5%)	0 (0.00%)	0 (0.00%)			
Additional diagnosis:						0.018	
Depression	0 (0.00%)	4 (10.0%)	NA	NA			
Depression & Past alcohol dependence	0 (0.00%)	1 (2.50%)	NA	NA			
Depression & Past panic disorder	0 (0.00%)	1 (2.50%)	NA	NA			
Depression & PTSD	0 (0.00%)	2 (5.00%)	NA	NA			
None	37 (100%)	31 (77.5%)	NA	NA			
Past alcohol dependence	0 (0.00%)	1 (2.50%)	NA	NA			
MDQ	1.11 (1.31)	9.32 (1.67)	NA	NA		<0.001	
Altman Mania (pre):					0.001	0.24	1
Mania	0 (0.00%)	3 (8.11%)	5 (27.8%)	5 (31.2%)			
None	37 (100%)	34 (91.9%)	13 (72.2%)	11 (68.8%)			
QIDS Depression (pre):					<0.001	<0.001	0.043
None	36 (97.3%)	18 (48.6%)	4 (22.2%)	1 (6.25%)			
Mild	1 (2.70%)	17 (45.9%)	5 (27.8%)	6 (37.5%)			
Moderate	0 (0.00%)	2 (5.41%)	8 (44.4%)	4 (25.0%)			
Severe	0 (0.00%)	0 (0.00%)	0 (0.00%)	5 (31.2%)			
Very severe	0 (0.00%)	0 (0.00%)	1 (5.56%)	0 (0.00%)			
Altman Mania (post):							1
Mania	0 (.%)	0 (.%)	4 (22.2%)	3 (20.0%)			
None	0 (.%)	0 (.%)	14 (77.8%)	12 (80.0%)			
QIDS Depression (post):							0.731
None	0 (.%)	0 (.%)	4 (22.2%)	1 (6.67%)			
Mild	0 (.%)	0 (.%)	7 (38.9%)	8 (53.3%)			
Moderate	0 (.%)	0 (.%)	4 (22.2%)	4 (26.7%)			
Severe	0 (.%)	0 (.%)	3 (16.7%)	2 (13.3%)			
Handedness:						0.757	
Right	32 (86.5%)	32 (80.0%)	NA	NA			
Ambidext	0 (0.00%)	1 (2.50%)	NA	NA			
Left	5 (13.5%)	7 (17.5%)	NA	NA			
# Behav. days	46.7 (3.63)	44.9 (6.61)	NA	NA		0.146	
# Behav. days (pre)	NA	NA	11.8 (6.72)	12.4 (5.04)			0.771
# Behav. days (post)	NA	NA	24.5 (7.85)	28.5 (9.32)			0.182
Has longitudinal data: Yes	37 (100%)	38 (95.0%)	19 (100%)	16 (100%)	0.762	0.494	1
Has FMRI data: Yes	37 (100%)	40 (100%)	13 (68.4%)	9 (56.2%)	<0.001	1	0.696
Day time difference (h) between longitudinal session	3.55 (1.19)	3.68 (1.16)	2.60 (1.25)	2.71 (1.39)	0.002	0.62	0.806
Most common longitudinal session time of day:					0.61	0.552	1
Afternoon	9 (24.3%)	12 (31.6%)	3 (15.8%)	2 (12.5%)			
Evening	24 (64.9%)	19 (50.0%)	14 (73.7%)	13 (81.2%)			
Morning	4 (10.8%)	6 (15.8%)	2 (10.5%)	1 (6.25%)			
Night	0 (0.00%)	1 (2.63%)	0 (0.00%)	0 (0.00%)			

Statistical tests are two-tailed *p*-values and refer to comparisons between the two groups of participants with low or high MDQ scores (‘Low vs. high MDQ’) and between the two groups of patients with BD randomized to lithium or placebo (‘Lith vs. pla’). Values are the mean and standard error of the mean. For the patients with BD, comorbid disorders were not measured. Note that in the low and high MDQ groups, diagnoses were only based on SCID, not on a full clinical examination. Participants completed weekly self-report scales of symptoms of mania (Altman) and depression (QIDS) at baseline (pre) and post assignment to lithium or placebo. The average scores pre (baseline) and post lithium were here categorized according to standard cut-offs (Altman: <6 for no mania, QIDS: 1–5: no depression, 6–10: mild depression, 11–15: moderate depression, 16–20: severe depression, 21–27: very severe depression). In short, lithium vs. placebo did not affect ratings of mania and depression, in line with the groups recruited here being outside major mood episodes requiring immediate treatment (see Supplementary Fig. S3 for time course of ratings). *# Behav. Days* number of days of behavioural data available (20 trials per day), *# Behav. Days (pre)* number of days in the baseline phase for the patients with BD, *# PANAS days* number of days with mood scores (PANAS, positive affect negative affect scale, short form) available, *MDQ* Mood disorder questionnaire, *Has longitudinal data: Yes* percentage of participants from whom longitudinal data (i.e., sessions at home) were available. Diagnoses: *BD-I* bipolar I disorder, *BD-II* bipolar II disorder, *BDNOS* bipolar disorder not otherwise specified, *PTSD* post traumatic stress disorder.

**Table 2:** Medication in patients with BD.

	Bipolar lith	Bipolar pla
# Participants	*N* = 19	*N* = 16
Medication:		
None	5 (8.93%)	3 (9.68%)
Atypical antipsychotic	16 (28.6%)	6 (19.4%)
Benzodiazepine	2 (3.57%)	1 (3.23%)
Beta blocker	1 (1.79%)	0 (0.00%)
Mood stabilizer	5 (9.80%)	3 (10.7%)
NA and DA reuptake inhibitor	1 (1.79%)	0 (0.00%)
Nonbenzodiazepine	1 (1.79%)	1 (3.23%)
Sedative	1 (1.79%)	0 (0.00%)
SNRI	1 (1.79%)	0 (0.00%)
SSRI	20 (35.7%)	12 (38.7%)
Tetracyclic antidepressant	2 (3.57%)	2 (6.45%)
Tricyclic antidepressant	0 (0.00%)	3 (9.68%)
Typical antipsychotic	1 (1.79%)	0 (0.00%)

At baseline, most patients were on stable doses of different medications, categorized here as: atypical antipsychotics (quetiapine, olanzapine, aripiprazole, risperidone, amisulpiride), benzodiazepine (clonazepam, lorazepam, diazepam), beta blocker (propranolol), mood stabilizer (valproate, lamotrigine), noradrenaline (NA) and dopamine (DA) reuptake inhibitor (buproprion), nonbenzodiazepine (zopiclone), sedative (promethazine), serotonin and noradrenaline reuptake inhibitor (SNRI, venlafaxine), SSRI (selective serotonin reuptake inhibitor (sertraline, citalopram, fluoxetine), tetracyclic antidepressant (mirtazapine), tricyclic antidepressant (dosulepin, lofepramine, amitriptyline), typical antipsychotic (stelazine, haloperidol).

### General performance

Participants completed longitudinal daily behavioural test sessions at home, consisting of 20 trials of a gambling task and mood self-reports. In the task (Fig. 1A), participants needed to choose repeatedly between two gambles (wheels of fortune), considering the probabilities of winning or losing points and the number of points that could be won or lost. Participants in all groups performed the task well (Fig. 1B), selecting options with higher values more frequently.

### Risk taking (avoidance of potential losses)

**Fig. 2:**
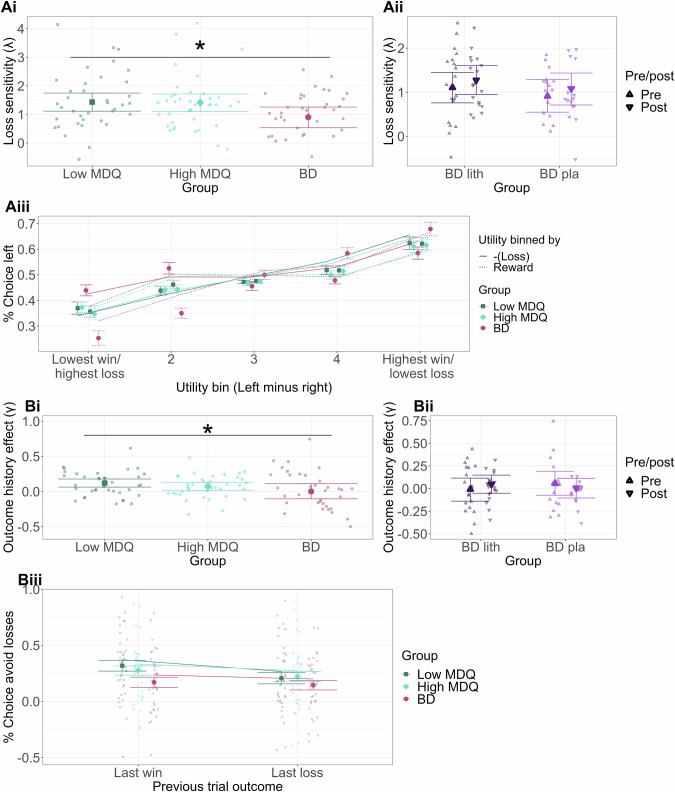
Group differences in longitudinal behaviour and mood. **A** Loss sensitivity. **Ai** Decreased loss sensitivity (λ, avoidance of potential losses) with bipolar disorder gradient, particularly for patients with BD. **Aii** Lithium (vs. placebo) did not affect loss sensitivity (group [lithium/placebo] * time [pre, i.e. baseline/post] interaction). **Aiii** Illustration of sensitivity of choices to loss/reward utility – as utility increases for the left compared to the right option, participants are more likely to choose the left option. For low/ high MDQ participants, this increase in choice probability is similar for the reward or loss dimension. In contrast, patients with BD show decreased sensitivity to losses vs. rewards (the loss curve is shallower. **B** Outcome history (i.e. adaptation of risk taking to past outcomes; avoidance of potential losses after a win [rather than loss] on the previous trial). **Bi** The outcome-history model parameter (γ) differed between the groups, with low MDQ participants showing the most and the patients with BD showed the least outcome history effects. **Bii** Lithium (vs. placebo) did not affect outcome history effects. **Biii** After a win vs. a loss on the previous trial (‘last win’/ ‘last loss’), low MDQ participants avoided losses more, while this was reduced with the MDQ gradient, so that patients with BD did not adapt their choices to past trial outcomes. A full list of comparisons of parameters for the groups is shown in Supplementary Tables S4 (longitudinal data) and Supplementary Table S6 (fMRI session data). Relationships between parameters measured longitudinally over weeks or in the lab during the fMRI session are shown in Supplementary Table S7. ii) and iii) show conditional effects from regression models, roughly equivalent to means, controlling for regressors of no interest. Lines in **Aiii** and **Biii** show the choices predicted by the model. Participant numbers: low MDQ: 37, high MDQ:40, BD lithium: 19, BD placebo: 16.

To test whether sensitivity to (i.e. avoidance of) potential losses vs. wins when gambling was reduced with a bipolar disorder gradient (low MDQ ≤high MDQ ≤patients with BD), we built a stochastic decision-making model that described participants’ choices as being based on the reward and loss utilities of the two options while allowing for individual differences in how people made decisions (see Supplementary Table S2 for model comparisons; model accuracy: 71%). The model captured participants’ sensitivity to losses (vs. wins) as a parameter (λ). We found that the higher the bipolar disorder gradient, the lower the sensitivity to losses vs wins (Fig. 2Ai, Supplementary Table S4A, mean = −0.27, 95% CI = [−0.49; −0.05]). This was driven mainly by a step change decrease in the group of patients with BD compared to the low/high MDQ groups, rather than a continuous linear relationship (Supplementary Table S4A for group comparison and continuous measure of mania symptoms across all groups). Lithium vs. placebo did not affect this (Fig. 2Aii, Supplementary Table S4B). To illustrate the effect in a model-free way, we plotted the sensitivity of choices to the win or loss dimensions (i.e., steepness of the curve, Fig. 2Aiii). This revealed that the difference between groups (group*win/loss dimension* utility bin: mean = 0.33, 95% CI = [0.06; 0.61]) is driven by both an increased sensitivity to wins (group*utility bin: mean 0.24, 95% CI = [0.08; 3.99]) and a decreased sensitivity to losses (group* utility bin: mean = −0.15, 95% CI = [−0.30; −0.01]) with the bipolar disorder gradient. Alternative computational models in Supplementary Table S5.

### Outcome history effects

We next analysed how participants adapted their risk taking across trials based on win or loss outcomes in the previous trial (‘outcome history effect’). In the computational model, outcome history effects were captured as a parameter (γ) that described to what extent participants were more sensitive to (i.e. avoidant of) potential losses after a win on the previous trial. We found that the bipolar disorder gradient reduced outcome history effects (Fig. 2Bi, Supplementary Tables S4A, S5 mean = −0.05, 95% CI = [−0.11; −0.0003], showing also a continuous effect with mania symptoms across all groups, Supplementary Table S4A). This was not affected by lithium (Fig. 2Bii, Supplementary Tables S4B, S5). We can unpack this effect in the data without a model (Fig. 2Biii) by focusing on the most extreme loss utility bins (if the loss utility difference is small, it will not affect choices if it is taken slightly more or less into account). If people show no outcome history effect, their choices should not change depending on the last trial’s outcome. However, the low MDQ group in fact takes the loss dimension more into account after a previous trial win vs. loss (i.e. less likely to pick options with high potential loss and more likely to pick options with low potential loss). This effect decreases with the bipolar disorder gradient (group*last loss/win: mean = 0.02, 95% CI = [0.0008; 0.04]).

### Mood

Finally, an advantage of the behavioural data being collected at home was that we could relate daily mood ratings to task-based behaviour. As reported previously [3, 45] and similar to other studies [2, 46, 47] groups differed in their instability (standard deviation) of mood: The low MDQ group showed the lowest and the patients with BD the highest mood instability (positive PANAS: mean = 0.22, 95%CI = [0.11; 0.33]; negative PANAS: mean = 0.64, 95%CI = [0.45; 0.83], Supplementary Table S8A, Supplementary Fig. S4A). Lithium did not affect instability when using our measure of standard deviation here (Supplementary Table S8B, Supplementary Fig. S4B), though note that using a measure of Bayesian volatility, lithium has been found to increase volatility of positive mood [3]. Across all groups, happiness VAS at the end of each session, compared to before was increased by overall (summed across the whole session) reward and decreased by loss outcomes (mean = 0.42, 95% CI = [0.31; 0.52]), similar to previous reports [48, 49]. However, this did not differ by bipolar disorder gradient (mean = −0.06, 95% CI = [−0.15, 0.03], Supplementary Table S8C). While mood instability differed between the groups, the impact on behaviour was distinct, with mood instability affecting the choice noisiness (the more unstable the mood, the more random the choices), without clearly affecting either loss sensitivity or outcome history effects (Supplementary Fig. S4D). The relationship between mood (PANAS) on the day of testing (rather than an overall measure of instability) and behaviour was not robust (Supplementary Table S8D). An exploratory analysis found that in the BD group, positive mood (PANAS) before the session led to reduced choice noisiness (Supplementary Fig. S4C, stats on the regression interaction term BD gradient x PANAS predicting choice noisiness: mean: 0.18, 95%CI: [0.01; 0.35]).

### Neural results

**Fig. 3:**
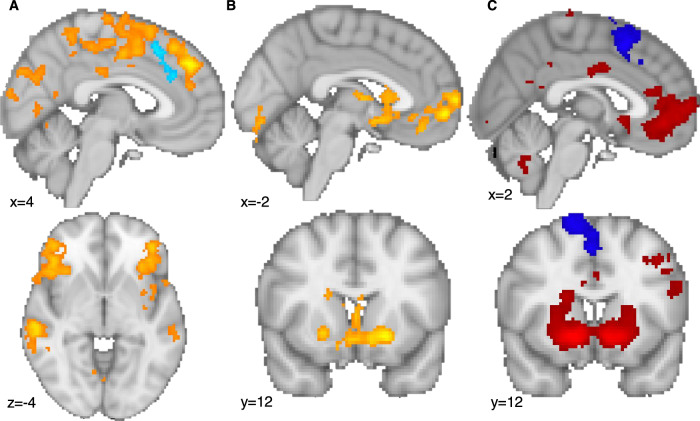
Neural activity during gambling. **A** At the time of the decision a wide network of areas activated with relative (chosen minus unchosen) reward utility (orange), while loss relative utility activated the anterior cingulate cortex (blue). **B** At the time of the decision, the last trial’s outcome (points won or lost) activated areas including vmPFC and ventral striatum (orange). **C** At the time of the outcome (win or loss received), the outcome (points won or lost) activated areas including vmPFC, FPm, and ventral striatum (red/orange) and deactivated the pre-supplementary area. All results are cluster-corrected at *p* < 0.05, two-tailed, with inclusion cut-off z > 2.3. See Supplementary Table S9 for the full list of results. Data were combined across both volunteer groups (low and high MDQ). Participant numbers: low MDQ: 37, high MDQ: 40.

Neural data were available for 77 volunteers and 22 patients. Across volunteers, brain activations to reward and loss utility during decisions (Fig. 3A) and at the receipt of outcomes (Fig. 3C) activated brain evaluation networks, including ventromedial prefrontal cortex (vmPFC), ventral striatum, dorsal anterior cingulate cortex (dACC), insula (Supplementary Table S9). Next, we tested whether, related to the outcome history effect, there was brain activity when participants made a choice that correlated with the previous trial’s outcome. Indeed, we found that activity in a network including the ventral striatum, vmPFC and medial frontal pole (FPm) related to the outcome of the previous trial, i.e. increased activity the more positive (and less negative) the previous trial’s outcome (Fig. 3B, Supplementary Table S9).

**Fig. 4:**
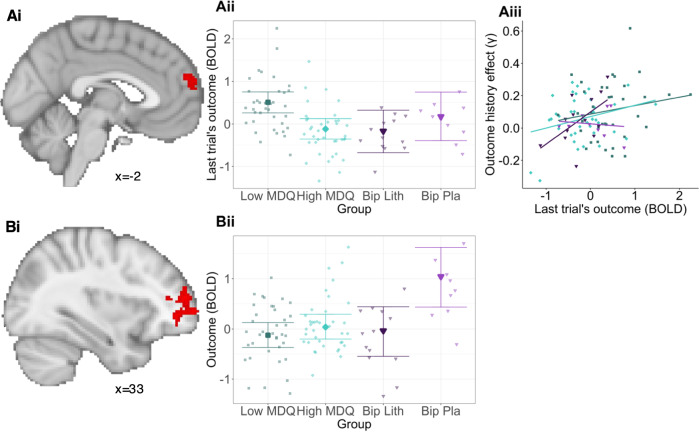
Group differences in brain signals. **A** Differences between the low and high MDQ groups for the outcome history effects. **Ai** Activation with last trial’s outcome at the time of the current trial’s decision differed between the low and high MDQ groups in the medial frontal pole (FPm; x = −10, y = 56, z = 16; p = 0.038, *n* = 77, cluster-corrected, Supplementary Table S10A. In the low MDQ group, the activation with the last trial’s outcome that is found across both groups (Fig. 3B) extends further dorsally. **Aii** This group difference was driven by the low MDQ group showing stronger activation than the high MDQ group in FPm (Figure shows conditional effects from regression model, roughly equivalent to means, controlling for regressors of no interest). There was no significant difference between activations comparing lithium and placebo groups (−0.30, 95%CI: [−0.73; 0.17]). **Aiii** This FPm activity correlated with the longitudinally measured outcome history parameter. Related whole-brain results shown in Supplementary Fig. S5. Colours match those of groups in B. **B** Exploratory whole-brain group differences in the patients with BD for gamble outcome signal (lithium vs. placebo). **Bi** Outcome related activity differed between the placebo and the lithium participants in an area including dorsolateral prefrontal cortex and lateral frontal pole (whole-brain cluster-corrected, Supplementary Table S10B). This effect is illustrated in **Bii**. Participant numbers: low MDQ: 37, high MDQ:40, BD lithium: 13, BD placebo: 9.

Next, we compared the low and high MDQ groups. Activity for the previous trial’s outcome was higher for the low MDQ vs high MDQ group in FPm (Fig. 4Ai–ii, Supplementary Table S10, *p* = 0.038, whole-brain cluster corrected). In other words, while all participants showed activity in vmPFC/FPm, in low MDQ participants the cluster extended further into FPm. Moreover, there was a correlation between the neural signal for the previous trial’s outcome and the behavioural outcome history effect: the stronger the activity for the last trial’s outcome in this area, the stronger the behavioural outcome history effect (Fig. 4Aiii, r = 0.24, *p* = 0.017, partial correlation after correction for control variables and group; without correction: r = 0.28, *p* = 0.005; test performed as robust regression, controlling for outliers: 95% Bayesian CI = [0.03; 1.52]). Lithium vs. placebo participants’ activity did not differ in this area (mean = 0.64, 95% CI = [−0.23; 1.44]).

As exploratory analyses (due to low sample sizes in BD groups for the MRI scan), we next compared lithium vs. placebo treatment at the whole-brain level. We found that patients receiving placebo had stronger activity related to the outcome of gambles in an area spanning dorsolateral prefrontal cortex (dlPFC, area 46) and lateral frontal pole (Fig. 4B, Supplementary Table S10B, *p* = 0.009). We also tested whether the gamble outcome activation related to the behavioural outcome history effect, finding that interestingly it did (Supplementary Fig. S5), in an mFP area overlapping with the area of group differences identified above, though not in the dlPFC area of group differences between lithium and placebo (Fig. 4A).

## Discussion

We designed a study to test the computational and neural correlates of adaptations of risk-taking to gains and losses in bipolar disorder (BD), in risk of bipolar disorder and treatment with lithium. We included participants along a gradient of bipolar disorder ranging from volunteers with low risk of BD (low MDQ group), to volunteers with high risk of BD, to patients with diagnosed BD. In the patients, we tested the effect of lithium treatment in a placebo-controlled double-blind design. We measured how much participants adapted their risk-taking following reward outcomes in a risky decision-making task (‘outcome history effects’). We measured behaviour both longitudinally over up to 50 days and during a brain imaging (FMRI) session. We found that the low MDQ group showed an ‘outcome history effect’. Specifically, after a win on a trial, they were more risk averse (avoiding potential losses). This was reduced across the bipolar disorder gradient (lowest risk aversion adaptation in patients with BD). Neurally, outcome history was related to the representation of past information in a large network including ventromedial prefrontal cortex (vmPFC) and medial frontal pole (FPm). In low MDQ volunteers, this brain signal extended further dorsally into FPm compared to the high MDQ scorers and this was correlated with risk adaption behaviour.

Decreased loss sensitivity and reward hypersensitivity have been suggested as central to BD [39, 40, 50, 51] and may drive risky or impulsive decision making. Our findings of decreased sensitivity to potential losses (vs wins) with BD gradient are in agreement with this. This effect showed a step change between the volunteer and the patient groups, rather than a continuous effect across the gradient. Then, we went further looking at adaptation of risk taking to past outcomes. We found that volunteers with presumed low risk of bipolar disorder (low MDQ) showed sequential dependencies between their choices and previous trials’ outcomes, avoiding potential losses after a win on the previous trial (‘outcome history effect’), as similarly recently reported in a go/no go decision-making task [52]. This was not strictly rational in our task since outcomes for gambles across trials were independent [10]. However, this kind of behaviour observed in the lab may be functionally appropriate in more naturalistic environments [38, 53–55] and thus reflect prior beliefs participants have about reward distributions (e.g. non-independence between trials). For example, in natural environments, which are experienced continually rather than in discrete trials and in which different types of rewards (e.g. food, water) need to be accumulated or a homeostatic setpoint needs to be reached, it would make sense to adapt behaviour according to previous outcomes [56–60]. The influence of past losses (vs wins) was lower in the high vs low MDQ group and lowest in patients with BD (i.e. the pattern showed a continuous gradient, also captured as a linear relationship to mania scores across all groups, rather than a step change from volunteers to patients). Reduced homeostatic behaviour of this kind could lead to unstable moods since in the healthy population mood has been found to be regulated through behaviour [8]. Relatedly, in patients with BD, purposefully regulating behaviour during the prodromal periods has been shown to reduce the risk of relapse [61]. However, in our study, links between ratings of mood and behaviour were weak and so this suggestion remains speculative. Future studies could measure mood over longer timescales, more frequently than done here and using a more naturalistic task [62, 63]. We also note that our findings diverge from previous findings [4] of a stronger impact of previous rewards (and associated emotions) on the perception of outcomes in a study including a participant sample not specifically selected for BD diagnosis or risk of bipolar disorder, but completing the Hypomanic Personality scale [64] after the task.

We focused on whole-brain analyses for the low/high MDQ volunteer sample due to the larger sample size compared to the patient study. Decision-making and the processing of outcomes produced a typical pattern of activation [12, 65–67] in areas including dorsal anterior cingulate cortex, striatum and vmPFC. However, there were no group differences in any of these signals, matching our behavioural results of an absence of differences in general ability to make decisions or sensitivity to rewards vs. losses per se in the low vs. high MDQ groups. We next looked for brain activity related to the modulation of risk taking with ‘outcome history’. We found that at the time when people made decisions, there was activity representing the last trial’s outcome in an area spanning vmPFC to FPm. This is similar to previous findings in a learning context of between-trial activities [14, 44, 68]. This gamble outcome activation was related to the behavioural outcome history effect across participants. This signal extended more dorsally into FPm in low MDQ volunteers. Furthermore, the stronger this signal, the stronger the modulation of risk taking by outcome history. As such the influence of outcomes on decision making may be a feature of risk for bipolar disorder which involves the FPm. This adds to previous work linking BD to changes in reward related signals in ventral striatum and OFC [19, 69] and changes in connectivity between striatum and PFC [17, 18]. In this region, lithium did not affect brain activity, suggesting that its mechanism of action may not involve direct modulation of vmPFC value weighting.

In an exploratory analysis, we compared the brain activity of patients with BD randomised to lithium or placebo. Patients given placebo showed larger outcome-related activity in dorsolateral prefrontal cortex. Yet at the same time, lithium did not change behaviour. dlPFC signalling has largely been associated with regulation of mood and reward-related behaviour. Previous work in bipolar disorder has showed altered patterns of both vmPFC and dlPFC activity. In particular, Mason et al. [17] reported that while controls activated dlPFC more to rewards of high probability, patients with bipolar disorder showed greater dlPFC to low probability (more risky) rewards. As such, our preliminary findings suggest that lithium may modulate a key component of frontostriatal circuitry important for effective decision making. Previous work in healthy volunteers also reported an effect of lithium on reward related signals in the ventral striatum which wasn’t detected in the current study [70].

The current work has a number of limitations. Our sample size was low for the comparison between lithium and placebo fMRI responses, which may have affected our statistical power for key comparisons. It is also relevant that we saw no effect of lithium on the clinical questionnaires included in this study. However, this is consistent with the characteristics of the sample recruited here, where current symptoms were largely residual (i.e. outside of an acute episode). Furthermore, lithium is largely used for relapse prevention rather than acute treatment of mania or depression [71] which could not be tested in the short timescale of the current investigation. Data across a large number of tasks and measures were also completed as part of these studies, and analysis is still ongoing. These complete results may shed light on the overall effects of bipolar disorder risk and treatment on different facets of mood and cognition. While we pre-registered our lithium trial (2014-002699-98), we did not pre-register our specific hypotheses for this part of the analysis. While we found an expected value signal (chosen minus unchosen value) in a typical ‘negative value’ network including the dACC, we did not find a ’positive value’ signal in a typically expected area like the vmPFC. This is unlikely to be due to signal drop out as vmPFC showed activation with reward outcome and an outcome history signal at the time of choice. This result is reminiscent of our previous findings [14], where it was interpreted as possibly due to the integration of an aversive dimensions (there: effort) with reward, rather than only integrating two positive dimensions (e.g. reward probability and reward magnitude). Similarly, here, participants were faced with a negative dimension, i.e. monetary loss.

Our results highlight the importance of considering rewarded decision-making and related neural activity to understand symptoms of bipolar disorder and the stabilising effects of lithium.

## Supplementary information

Supplements

## Data Availability

Code and anonymized (behaviour, selected demographics, brain activity from regions of interest) data are available on osf.io, 10.17605/OSF.IO/YCHBF. Group-level whole-brain maps are available on the same osf.io directory and on neurovault: https://identifiers.org/neurovault.collection:19030.

## Code availability

Code and anonymized (behaviour, selected demographics, brain activity from regions of interest) data are available on osf.io, 10.17605/OSF.IO/YCHBF. Group-level whole-brain maps are available on the same osf.io directory and on neurovault: https://identifiers.org/neurovault.collection:19030.
