# Supplementary material for: Neural signatures of risk-taking adaptions across health, bipolar disorder, and lithium treatment
Source: Mol Psychiatry. 2025 Jan 29;30(7):2955–65. doi: 10.1038/s41380-025-02900-w (PMC12185344; doi:10.1038/s41380-025-02900-w)
Supplement: Supplementary file 1 — Supplements [file 41380_2025_2900_MOESM1_ESM.pdf]

Supplements for Neural signatures of risk-taking adaptions across health, bipolar disorder, and lithium treatment

Jacqueline Scholl, Priyanka Panchal, Natalie Nelissen, Lauren Z Atkinson, Nils Kolling, Kate EA Saunders, John Geddes, Matthew FS Rushworth, Anna C Nobre, Paul J Harrison, Catherine J Harmer

	$t.\beta\_mean$	$t.\lambda\_mean$	$t.\gamma\_mean$	$t.\beta\_sd$	$t.\lambda\_sd$	$t.\gamma\_sd$	$f.\beta\_mean$	$f.\lambda\_mean$	$f.\gamma\_mean$	$f.\beta\_sd$	$f.\lambda\_sd$	$f.\gamma\_sd$
$t.\beta\_mean$		0.018	-0.095	0.150**	0.024	-0.077	0.875***	-0.129**	-0.088	-0.213***	0.204***	0.008
$t.\lambda\_mean$	0.018		0.064	-0.000	-0.055	0.024	-0.307***	0.962***	0.060	0.318***	0.427***	0.592***
$t.\gamma\_mean$	-0.095	0.064		-0.045	-0.045	-0.042	-0.103*	0.061	0.911***	0.063	-0.045	0.059
$t.\beta\_sd$	0.150**	-0.000	-0.045		-0.078	-0.026	0.107*	-0.023	-0.055	0.178***	-0.037	-0.011
$t.\lambda\_sd$	0.024	-0.055	-0.045	-0.078		-0.003	0.078	-0.041	-0.055	0.047	0.641***	-0.099*
$t.\gamma\_sd$	-0.077	0.024	-0.042	-0.026	-0.003		-0.083	0.039	-0.004	-0.027	-0.027	0.428***
$f.\beta\_mean$	0.875***	-0.307***	-0.103*	0.107*	0.078	-0.083		-0.423***	-0.094	-0.232***	0.057	-0.212***
$f.\lambda\_mean$	-0.129**	0.962***	0.061	-0.023	-0.041	0.039	-0.423***		0.055	0.379***	0.393***	0.575***
$f.\gamma\_mean$	-0.088	0.060	0.911***	-0.055	-0.055	-0.004	-0.094	0.055		0.090	-0.064	0.069
$f.\beta\_sd$	-0.213***	0.318***	0.063	0.178***	0.047	-0.027	-0.232***	0.379***	0.090		0.198***	0.177***
$f.\lambda\_sd$	0.204***	0.427***	-0.045	-0.037	0.641***	-0.027	0.057	0.393***	-0.064	0.198***		0.216***
$f.\gamma\_sd$	0.008	0.592***	0.059	-0.011	-0.099*	0.428***	-0.212***	0.575***	0.069	0.177***	0.216***	

Computed correlation used pearson-method with listwise-deletion.

Table S1. Parameter recovery, longitudinal data (related to main text methods ‘Computational models’)**.** Parametric (Pearson) correlations between ground-truth (‘t.’) and fitted (‘f.’) parameters. We simulated 400 participants with mean (‘_mean’) and standard (‘_sd’) from which choices for individual sessions of 20 trials were then generated (see supplementary methods [2A]). Simulated participants provided 47-50 sessions of data (50%-100% range of participants). Parameters: inverse temperature (β), sensitivity to loss utility (λ) and change in loss sensitivity after prev. trial win vs. loss (γ). Results show that recovery for mean parameters was very good (correlations between true and corresponding fitted, all >0.68). However, recovery for standard deviations was poor (e.g. γ_sd: r=0.24). Given this, we decided not to analyse group differences in standard deviations. Neither did we therefore attempt more complex models of variations of parameters across time, such as a volatility model (1).

Model type	inv. temp	variance	skew	outcome hist	reward dist.	loss dist.	loss weight	AIC
M3	x	x	x	x				0
M3	x	x	x					99
M3	x	x	x					133
M3	x	x	x					215
M2	x			x[1]	x	x		3972
M2	x				x	x		4052
M2	x			x[2]	x	x		4131
M3	x		x					5029
M2	x				x[3]	x[3]		6126
M1	x			x			x	6869
M1	x						x	6875
M2	x					x		6972
M2	x				x			7127
M3	x	x						7412
M1/2/3	x							11431

Table S2. Model comparison with Akaishi Information Criterion (AIC) (related to main text methods 'Computational models'). We compared three types of models. M1 (model used in main manuscript): decision variables as weighted combination of reward and loss (methods in main text); M2: decision variable containing exponential scaling of reward and loss magnitudes (supplementary methods [2B]); M3: weighted combination expected value, variance and skew (supplementary methods [2B]). Each of these models was run with different parameters being included or excluded ('x' in the table indicates inclusion) – parameter abbreviations: inv. temp = inverse temperature; outcome hist = outcome history; reward dist.= exponential distortion of reward magnitude; loss dist. = exponential distortion of loss magnitude. Annotations: [1]Model of type M2, with outcome history included as linear weighing of the loss expected value; [2]Model of type M2, with outcome history included in the exponential weighing; [3]Model of type M2, with a shared parameter for exponential distorting of reward and loss magnitudes. AIC values are shown relative to the best fitting model, higher numbers indicate worse fit. Importantly, for all types of models, there is a model including outcome history that provides the best fit. While the model presented in the paper (M1) showed not the best fit to the data, it was retained it for ease of meaning of parameters (e.g. sensitivity to potential losses vs. sensitivity to skew) and analogy to previous decision making studies. Potentially, in the present study, the reason that a model with variance and skew provides a better fit is because total expected value of the two options was often very similar (Figure S1). However, we note, that the key behavioural findings of the paper, i.e. decrease in the outcome history effect with mood elevation gradient and decrease in loss aversion in BP are also captured in the both models M2 and M3, see Table S5. Though note that for the outcome history effect in M3, the 95%CI for the effect just about included zero [-0.0006;0.06], while the difference between the BD and low MDQ and high MDQ groups was significant). Parameter recovery for M2 and M3 type models in Table S3.

**M3**	t.invTemp	t.var weight	t.skew weight	t.outcome history	f.invTemp	f.var weight	f.skew weight	f.outcome history
t.invTemp		-0.058	-0.109*	-0.08	0.867***	-0.081	-0.145**	-0.131**
t.var weight	-0.058		0.027	0.026	-0.069	0.978***	0.01	0.049
t.skew weight	-0.109*	0.027		0.067	-0.196***	0.015	0.977***	0.106*
t.outcome history	-0.08	0.026	0.067		-0.082	0.017	0.058	0.907***
f.invTemp	0.867***	-0.069	-0.196***	-0.082		-0.081	-0.220***	-0.114*
f.var weight	-0.081	0.978***	0.015	0.017	-0.081		0.001	0.041
f.skew weight	-0.145**	0.01	0.977***	0.058	-0.220***	0.001		0.098
f.outcome history	-0.131**	0.049	0.106*	0.907***	-0.114*	0.041	0.098	

**M2**	t.invTemp	t.loss mag dist	t.rew mag dist	t.outcome history	f.invTemp	f.loss mag dist	f.rew mag dist	f.outcome history
t.invTemp		0.075	-0.046	-0.052	0.901***	0.099*	-0.091	0.006
t.loss mag dist	0.075		-0.029	-0.023	0.074	0.820***	0.112*	-0.009
t.rew mag dist	-0.046	-0.029		0.06	-0.057	0.312***	0.905***	0.082
t.outcome history	-0.052	-0.023	0.06		-0.06	0.019	0.071	0.914***
f.invTemp	0.901***	0.074	-0.057	-0.06		0.077	-0.135**	0.011
f.loss mag dist	0.099*	0.820***	0.312***	0.019	0.077		0.423***	0.046
f.rew mag dist	-0.091	0.112*	0.905***	0.071	-0.135**	0.423***		0.09
f.outcome history	0.006	-0.009	0.082	0.914***	0.011	0.046	0.09	

Table S3. Parameter recovery for alternative models (M2, M3, see Table S2 and supplementary methods [2B], related to main text methods 'Computational models')**.** Shown are only the parameter recovery results for participant-wise means, the standard deviations showed equally bad recovery as for M1 (Table S1).

A) Low MDQ, High MDQ, Bipolar disorder (BD) groups (baseline)			
**Group**	**Choice consist. (β)**	**Loss sensitivity (λ)**	**Outcome history effect (γ)**
**3 group gradient**	-0.154 [-0.5698 0.2498]	-0.269 [-0.4868 -0.0519]	-0.053 [-0.1104 -3e-04]
**High vs low MDQ**	-0.42 [-1.15 0.36]	-0.01 [-0.41 0.39]	-0.05 [-0.11 0.03]
**BD vs high MDQ**	0.14 [-0.62 0.9]	-0.5 [-0.93 -0.08]	-0.07 [-0.19 0.05]
**BD vs low MDQ**	-0.3 [-1 0.52]	-0.52 [-0.94 -0.08]	-0.12 [-0.24 0]
**BD**	5.84 [5.24 6.49]	0.89 [0.56 1.23]	0 [-0.11 0.1]
**High MDQ**	5.71 [5.13 6.29]	1.4 [1.12 1.69]	0.07 [0.02 0.12]
**Low MDQ**	6.13 [5.55 6.71]	1.41 [1.13 1.72]	0.11 [0.06 0.16]
**Day**	-0.555 [-0.763 -0.339]	-0.239 [-0.329 -0.155]	-0.03 [-0.062 0.002]
**Mania**	-0.208 [-0.4911 0.0728]	-0.087 [-0.2375 0.0625]	-0.039 [-0.072 -0.0056]

B) BD groups, pre/post * lithium/placebo			
**Group**	**Choice consist. (β)**	**Loss sensitivity (λ)**	**Outcome history effect (γ)**
**Lith/pla x pre/post**	0.193 [-0.9163 1.2654]	-0.01 [-0.5146 0.4884]	-0.11 [-0.2983 0.0797]
**Lith pre vs post**	0.36 [-0.49 1.2]	-0.18 [-0.53 0.16]	-0.06 [-0.19 0.08]
**Pla pre vs post**	0.17 [-0.78 1.09]	-0.17 [-0.54 0.21]	0.05 [-0.09 0.2]
**Lith vs pla (pre)**	0.76 [-0.42 1.83]	0.19 [-0.25 0.61]	-0.07 [-0.23 0.1]
**Lith vs pla (post)**	0.55 [-0.6 1.73]	0.2 [-0.22 0.62]	0.04 [-0.08 0.17]
**Lith (pre)**	6.36 [5.43 7.29]	1.09 [0.8 1.39]	-0.03 [-0.15 0.09]
**Placebo (pre)**	5.61 [4.61 6.6]	0.9 [0.58 1.23]	0.04 [-0.08 0.17]
**Lith (post)**	6 [5.19 6.88]	1.26 [0.97 1.55]	0.03 [-0.06 0.11]
**Placebo (post)**	5.44 [4.5 6.35]	1.06 [0.76 1.38]	-0.01 [-0.1 0.09]

Table S4. Computational modelling results (related to Figure 2). Computational model parameters for the longitudinal data. A) Comparison of the three groups (mood elevation gradient, ordered factors across low MDQ, high MDQ, patients with BD, at baseline, i.e. pre-randomization to lithium or placebo). The groups differed in their loss sensitivity (patients with BD being least sensitive to losses) and outcome history effects (patients with BD being least sensitive to past trial outcomes). How participants performed the task changed over time (effect of 'Day'), in particular they became more random (lower inverse temperature) and less sensitive to potential losses (loss sensitivity). When repeating the analyses, but omitting the 5 participants from the high MDQ group that were given a BD diagnosis (Table 1) during the intake interview, results remained broadly the same. For loss sensitivity, we find the same results as before (-0.26; 95% CI: [-0.48; -0.03]). For the outcome history effect, the group effect is not quite significant anymore, but numerically very close to the previous finding (-0.05; 95%CI: [-0.11; 0.0006]). When repeating the analyses and including as additional regressor for each session the time (days) since the previous session, we found numerically very similar results (Loss sensitivity: -0.25; 95%CI: [-0.47;-0.03]; outcome history: -0.0586, 95%CI: [-0.105; 0.0026]). Repeating the analyses and replacing group assignment by the Altman Mania score ('Mania', continuous measure, available for all but one participant in the BD group [later assigned to lithium] and 3 in the high MDQ group), outcome history remains significant, while loss sensitivity is no longer significant (though trend in the same direction as considering group). B) Comparison for the effect of lithium vs. placebo in hierarchical models (Main text Methods, section 'Model fitting', term of interest is the interaction drug (lithium/placebo) * time (pre/post)). No significant group differences were found. Values are means and 95% Bayesian Credible Intervals; for comparisons between groups, significance is defined as 95% intervals not including zero. All estimates were obtained from hierarchical regression models (Main text Methods, section 'Model fitting').

A) M3: Expected value, variance, skew				
**Ai) M3: Low MDQ, High MDQ, Bipolar disorder (BD, baseline) groups**				
**Group**	**Choice consist. (β)**	**Var Weight**	**Skew Weight**	**Outcome history effect**
**3 group gradient**	-0.114 [-0.4411 0.2132]	0.108 [0.0072 0.2066]	0.047 [-0.0497 0.145]	0.027 [-6e-04 0.0556]
**High vs low MDQ**	0.05 [-0.53 0.62]	0.01 [-0.17 0.19]	0.04 [-0.13 0.22]	0.01 [-0.03 0.04]
**BD vs high MDQ**	-0.26 [-0.82 0.42]	0.21 [0.02 0.42]	0.06 [-0.13 0.27]	0.06 [0 0.11]
**BD vs low MDQ**	-0.21 [-0.81 0.42]	0.22 [0.02 0.42]	0.1 [-0.09 0.31]	0.06 [0.01 0.12]
**BD**	2.62 [2.12 3.13]	0.23 [0.07 0.38]	0.1 [-0.05 0.26]	0.01 [-0.04 0.06]
**High MDQ**	2.87 [2.43 3.33]	0.02 [-0.12 0.14]	0.04 [-0.08 0.17]	-0.04 [-0.07 -0.02]
**Low MDQ**	2.83 [2.36 3.27]	0.01 [-0.12 0.14]	0 [-0.14 0.13]	-0.05 [-0.08 -0.02]
**Day**	-0.133 [-0.297 0.033]	0.047 [0.015 0.08]	0.108 [0.07 0.147]	0.014 [-0.001 0.029]
**Aii) M3: BD groups, pre/post * lithium/placebo**				
**Group**	**Choice consist. (β)**	**Var Weight**	**Skew Weight**	**Outcome history effect**
**Lith/pla x pre/post**	-0.273 [-1.1636 0.6362]	0.152 [-0.0605 0.364]	-0.217 [-0.4738 0.0465]	-0.006 [-0.1042 0.0895]
**Lith pre vs post**	-0.28 [-0.96 0.4]	0.16 [0.02 0.32]	-0.04 [-0.22 0.14]	0.02 [-0.05 0.09]
**Pla pre vs post**	-0.01 [-0.72 0.7]	0.01 [-0.14 0.18]	0.18 [-0.03 0.37]	0.02 [-0.05 0.1]
**Lith vs pla (pre)**	0.61 [-0.23 1.5]	0.09 [-0.12 0.3]	-0.11 [-0.39 0.16]	0 [-0.09 0.08]
**Lith vs pla (post)**	0.89 [-0.03 1.81]	-0.06 [-0.25 0.12]	0.1 [-0.2 0.43]	0 [-0.06 0.06]
**Lith (pre)**	2.83 [2.14 3.49]	0.24 [0.1 0.39]	-0.03 [-0.22 0.16]	0.01 [-0.05 0.07]
**Placebo (pre)**	2.22 [1.53 2.92]	0.15 [-0.01 0.3]	0.09 [-0.11 0.28]	0.02 [-0.05 0.08]
**Lith (post)**	3.11 [2.45 3.77]	0.08 [-0.05 0.2]	0.01 [-0.19 0.22]	-0.01 [-0.05 0.03]
**Placebo (post)**	2.22 [1.54 2.93]	0.14 [0 0.27]	-0.09 [-0.32 0.14]	-0.01 [-0.05 0.04]
**B) M2: exponential distortions of magnitudes**				
**Bi) M2: Low MDQ, High MDQ, BD (baseline) groups**				
**Group**	**Choice consist. (β)**	**Rew mag dist**	**Loss mag dist**	**Outcome history effect**
**3 group gradient**	-0.362 [-0.8268 0.1023]	0.046 [-0.0291 0.1216]	-0.078 [-0.1551 -1e-04]	0.023 [3e-04 0.0532]
**High vs low MDQ**	-0.12 [-1 0.76]	0.04 [-0.1 0.17]	0 [-0.13 0.13]	0.02 [0 0.04]
**BD vs high MDQ**	-0.48 [-1.3 0.43]	0.06 [-0.1 0.21]	-0.16 [-0.32 -0.02]	0.03 [-0.04 0.1]
**BD vs low MDQ**	-0.6 [-1.48 0.25]	0.1 [-0.06 0.26]	-0.16 [-0.31 -0.01]	0.05 [-0.02 0.12]
**BD**	7.12 [6.4 7.9]	1.34 [1.22 1.47]	1.32 [1.2 1.44]	0.04 [-0.03 0.1]
**High MDQ**	7.6 [6.91 8.34]	1.29 [1.19 1.39]	1.49 [1.39 1.58]	0.01 [-0.01 0.03]
**Low MDQ**	7.73 [7.01 8.47]	1.25 [1.15 1.34]	1.48 [1.39 1.57]	-0.01 [-0.03 0.01]
**Day**	-0.668 [-0.928 -0.409]	0.056 [0.027 0.085]	0.029 [-0.002 0.06]	0.012 [0.001 0.023]
**Bii) M2: BD groups, pre/post * lithium/placebo**				
**Group**	**Choice consist. (β)**	**Rew mag dist**	**Loss mag dist**	**Outcome history effect**
**Lith/pla x pre/post**	-0.073 [-1.2749 1.123]	-0.085 [-0.2576 0.0919]	-0.202 [-0.4042 3e-04]	0.054 [-0.1137 0.2272]
**Lith pre vs post**	-0.16 [-1.05 0.74]	0.01 [-0.11 0.12]	-0.07 [-0.21 0.08]	-0.01 [-0.12 0.11]
**Pla pre vs post**	-0.09 [-1.15 0.99]	0.09 [-0.03 0.23]	0.13 [-0.02 0.29]	-0.06 [-0.2 0.07]
**Lith vs pla (pre)**	0.83 [-0.4 2.01]	-0.02 [-0.18 0.15]	-0.09 [-0.36 0.2]	0.04 [-0.11 0.18]
**Lith vs pla (post)**	0.9 [-0.5 2.29]	0.07 [-0.13 0.27]	0.11 [-0.14 0.35]	-0.02 [-0.13 0.09]
**Lith (pre)**	7.55 [6.52 8.57]	1.29 [1.17 1.4]	1.26 [1.07 1.46]	0.02 [-0.08 0.12]
**Placebo (pre)**	6.71 [5.62 7.8]	1.3 [1.18 1.42]	1.36 [1.16 1.56]	-0.02 [-0.12 0.1]
**Lith (post)**	7.71 [6.64 8.76]	1.28 [1.15 1.42]	1.33 [1.17 1.5]	0.03 [-0.04 0.09]
**Placebo (post)**	6.81 [5.63 7.94]	1.21 [1.06 1.35]	1.22 [1.05 1.4]	0.05 [-0.04 0.12]

Table S5. Group comparisons for alternative models (M2, M3, see Table S2 and supplementary methods [2B]). A) In M3, instead of considering reward and loss expected values/ utility separately, we consider total expected value, variance and skew. Now, the previous (M1) decreased loss risk aversion with BD expressed

itself as increased preference for options with higher variance (see Figure S1 for relationship – high correlation- between risk of loss and variance). The mood elevation gradient is again linked to decreased adaptation across trials, here captured as decreased preference to options with high variance after win on previous trial (in M1: decreased avoidance of risk of losing). B) In M2, the model has parameters for the (exponential) distortion of reward magnitudes and loss magnitudes. Outcome history effects are captured as linear weighting of the loss expected value. Again, group differences captured are conceptually very similar to M1. As we could not fit a model (due to low trial numbers per session) including both the linear and exponential effects of loss sensitivity, future studies will need to be done to describe the specific shape of the increased loss sensitivity more precisely. Values are means and 95% Bayesian Credible Intervals; for comparisons between groups, significance is defined as 95% intervals not including zero.

Group	Choice consist. (β)	Loss sensitivity (λ)	Outcome history effect (γ)
**Lith (post)**	8.8 [7.08 10.52]	1.48 [0.92 2.03]	-0.07 [-0.31 0.17]
**Pla (post)**	6.58 [4.55 8.48]	1.58 [0.95 2.29]	-0.01 [-0.27 0.27]
**High MDQ**	7.06 [6.08 8.08]	1.76 [1.38 2.07]	0.13 [-0.02 0.27]
**Low MDQ**	7.34 [6.28 8.34]	1.73 [1.38 2.09]	0.12 [-0.03 0.25]
**4 Group diff (post)**	-0.002 [-0.74 0.67]	-0.059 [-0.278 0.157]	-0.051 [-0.141 0.039]
**Lith vs pla (post)**	2.23 [-0.3 4.95]	-0.1 [-0.96 0.75]	-0.06 [-0.42 0.28]
**High vs low MDQ**	-0.28 [-1.61 1.21]	0.03 [-0.46 0.53]	0.02 [-0.17 0.21]

Table S6. FMRI session computational parameters (related to Figure 2). Values are reported separately for each of the four groups (Bipolar participants on lithium ('Bip Lith'), bipolar participants on placebo ('Bip Pla'), healthy volunteers with low or high mood instability ('Low MDQ', 'High MDQ') as means and 95% Bayesian Credible Intervals (intervals not including zero are significant).

All estimates were obtained from linear regression models allowing correcting for age and gender. Group differences were computed across all four groups ('4 group diff'). Group differences are also reported separately comparing high and low mood instability participants ('High vs. low MDQ') and lithium vs. placebo participants ('Lith vs pla'). There were no significant group differences.

	β_mean	λ_mean	γ_mean	β_fmri	λ_fmri	γ_fmri
β_mean		-0.043	0.147	0.394***	-0.249*	-0.067
λ_mean	-0.043		0.101	-0.180	0.426***	0.178
γ_mean	0.147	0.101		-0.047	-0.034	-0.058
β_fmri	0.394***	-0.180	-0.047		-0.525***	-0.076
λ_fmri	-0.249*	0.426***	-0.034	-0.525***		0.193
γ_fmri	-0.067	0.178	-0.058	-0.076	0.193	

Computed correlation used pearson-method with listwise-deletion.

Table S7. Correlations between parameters from longitudinal and FMRI data (related to Figure 2). For all but one parameter, computational parameters derived from longitudinal measurements and those obtained during the FMRI scan correlate significantly. Only for the outcome history effect parameter (γ) are the correlations not significant. Correlations were computed using Pearson correlations across combined data from all four participant groups.

A) Low MDQ, High MDQ, Bipolar disorder (BD) groups (baseline)				
**Group**	**Positive PANAS (mean)**	**Negative PANAS (mean)**	**Positive PANAS (sd)**	**Negative PANAS (sd)**
3 group gradient	0.15 [-0.6434 0.9624]	2.61 [1.9555 3.2551]	0.221 [0.1118 0.3321]	0.642 [0.4558 0.8305]
High - low MDQ	0.16 [-1.29 1.67]	1.26 [-0.02 2.47]	0.35 [0.15 0.56]	0.73 [0.41 1.06]
BD - high MDQ	0.04 [-1.22 1.38]	2.1 [0.9 3.25]	0.1 [-0.12 0.34]	0.6 [0.23 0.95]
BD - low MDQ	0.2 [-1.11 1.52]	3.36 [2.18 4.45]	0.45 [0.22 0.68]	1.33 [0.94 1.71]
BD	6.76 [5.63 7.86]	4.94 [3.97 5.87]	1.15 [0.97 1.33]	1.13 [0.84 1.41]
High MDQ	6.73 [5.75 7.75]	2.83 [2.03 3.73]	1.05 [0.9 1.19]	0.53 [0.3 0.75]
Low MDQ	6.56 [5.41 7.69]	1.57 [0.73 2.53]	0.7 [0.56 0.86]	-0.2 [-0.43 0.05]
Day	-0.296 [-0.56 -0.049]	-0.007 [-0.139 0.119]	NA	NA

B) BD groups, pre/post * lithium/placebo				
**Group**	**Positive PANAS (mean)**	**Negative PANAS (mean)**	**Positive PANAS (sd)**	**Negative PANAS (sd)**
Lith/pla x pre/post	-0.274 [-1.5488 1.019]	0.264 [-0.8541 1.3526]	0.013 [-0.4098 0.4187]	-0.086 [-0.4867 0.3164]
Lith pre vs post	1.01 [-0.03 2.08]	0.86 [-0.01 1.72]	0.22 [-0.06 0.51]	0.1 [-0.19 0.36]
Pla pre vs post	1.3 [0.12 2.58]	0.58 [-0.41 1.59]	0.2 [-0.11 0.5]	0.18 [-0.12 0.46]
Lith vs pla (pre)	0.2 [-1.61 1.86]	-0.35 [-2 1.31]	0.06 [-0.43 0.49]	-0.2 [-0.65 0.25]
Lith vs pla (post)	0.49 [-1.59 2.51]	-0.62 [-2.47 1.27]	0.04 [-0.36 0.47]	-0.11 [-0.57 0.32]
Lith (pre)	7.92 [5.98 9.87]	6.19 [4.41 8.04]	1.17 [0.84 1.48]	0.88 [0.54 1.18]
Placebo (pre)	7.72 [5.78 9.7]	6.55 [4.62 8.35]	1.11 [0.77 1.45]	1.08 [0.75 1.39]
Lith (post)	6.89 [4.98 8.9]	5.33 [3.57 7.3]	0.95 [0.66 1.23]	0.78 [0.46 1.07]
Placebo (post)	6.43 [4.48 8.6]	5.97 [4.09 8.02]	0.91 [0.59 1.19]	0.89 [0.58 1.21]

C) Group differences in impact of gambling outcomes on mood			
**Regressor**	**Total gain**	**Total loss**	**Total gain minus loss**
Outcome->Mood change	0.843 [0.6303 1.0599]	0.942 [0.7177 1.1755]	1.026 [0.7737 1.286]
3 group gradient	0.12 [-0.0254 0.2764]	0.188 [0.0291 0.3525]	0.133 [-0.0037 0.2764]
3 group gradient x outcome	-0.083 [-0.2753 0.0951]	-0.146 [-0.3453 0.0576]	-0.123 [-0.3475 0.0926]

D) Impact of mood before the task on task behaviour			
**Regressor**	**Choice consist. (β)**	**Loss sensitivity (λ)**	**Outcome history (γ)**
totalPANAS (not ctr gr)	0.034 [-0.0835 0.1533]	0.007 [-0.0488 0.061]	-0.01 [-0.0396 0.0187]
totalPANAS (ctr gr)	0.03 [-0.0856 0.1478]	0 [-0.0548 0.053]	-0.017 [-0.0484 0.014]
totalPANAS (ctr interact)	-0.093 [-0.2803 0.086]	-0.007 [-0.0973 0.0834]	-0.022 [-0.0708 0.0239]
totalPANAS x 3gr gradient	0.157 [-0.0078 0.3185]	0.005 [-0.0666 0.0772]	0.005 [-0.0355 0.0438]
3 group gradient (ctr totalPANAS interact)	-0.095 [-0.5057 0.3175]	-0.268 [-0.4829 -0.0452	-0.059 [-0.1189 -0.0041]
posPANAS (not ctr gr)	0.03 [-0.0933 0.158]	-0.015 [-0.0719 0.0419]	0 [-0.0299 0.0306]
posPANAS (ctr gr)	0.009 [-0.0289 0.0491]	-0.009 [-0.0466 0.0287]	0.002 [-0.0367 0.0407]
posPANAS (ctr interact)	-0.093 [-0.2756 0.0794]	-0.017 [-0.1056 0.0673]	-0.001 [-0.047 0.0439]
posPANAS x 3gr gradient	0.177 [0.0059 0.3461]	0.002 [-0.0732 0.0782]	0.001 [-0.0417 0.0412]
3 group gradient (ctr posPANAS interact)	-0.166 [-0.5529 0.2334]	-0.265 [-0.4898 -0.038]	-0.056 [-0.1126 -0.0032]
negPANAS (not ctr gr)	-0.031 [-0.1628 0.0978]	-0.037 [-0.0992 0.0274]	0.019 [-0.0122 0.0505]
negPANAS (ctr gr)	-0.007 [-0.0487 0.0359]	-0.014 [-0.0576 0.0298]	0.045 [0.0025 0.0872]
negPANAS (ctr interact)	0.036 [-0.2153 0.2919]	-0.012 [-0.1293 0.1091]	0.071 [0.0039 0.1487]
negPANAS x 3gr gradient	-0.053 [-0.2339 0.1254]	-0.008 [-0.0952 0.0775]	-0.027 [-0.0747 0.0178]
3 group gradient (ctr negPANAS interact)	-0.114 [-0.5185 0.3209]	-0.232 [-0.4619 0.0057]	-0.071 [-0.1345 -0.0138]

Table S8. Group difference for mood (PANAS) mean and standard deviations and impact of task outcomes on momentary mood (VAS). A) Comparison of the three groups (mood elevation gradient, ordered factors across low MDQ, high MDQ, patients with BD [i.e. pre randomization to lithium or placebo]). The groups differed in variability (standard deviation (log scale) for positive and negative PANAS with patients with BD

showing the highest variability. Groups also differed in the mean values for negative PANAS. B) Comparison for the effect of lithium vs. placebo in hierarchical models (Main text Methods, section 'Model fitting', term of interest is the interaction drug (lithium/placebo) * time (pre i.e. baseline /post)). No significant group differences were found. C) In a regression predicting changes in mood rated on a visual analogue scale (VAS) post completing the daily task vs. pre, there was an overall effect that the higher the total reward, and the lower the total loss (i.e. more positive number), the more mood improves. However, this impact of task outcomes on mood was not affected by the mood elevation gradient ('3 Group x outcome'). Values are means and 95% Bayesian Credible Intervals; significance is defined as 95% intervals not including zero. All estimates were obtained from hierarchical regression models (Main text Methods, section 'Model fitting'). D) In separate regressions, we assessed the impact of mood behaviour the daily tasks on behaviour. Mood was measured as positive or negative PANAS or as the total PANAS (positive minus negative PANAS). The regressions were of the same form as throughout the paper (e.g. S4), additionally including mood and/or an interaction between mood and BD gradient. For completeness, we report the results here for regressions only including mood, not BD gradient ('not ctr gr'), controlling BD gradient in addition to mood ('ctr gr'), controlling for an interaction between mood and BD gradient ('ctr interact'). We report also the interactions between mood and BD gradient ('x 3gr gradient') and the result for BD gradient, controlling intearctions with mood ('ctr mood interact'). We find (figure S4C for illustration) that in the BD group, choice consistency is higher (i.e. less choice noisiness) when positive PANAS is higher. While there appears to be an impact of negative PANAS on outcome history, this only emerges when including the BD gradient as a regressor, making it difficult to interpret (one could speculate that a 'masking' effect is present because neg PANAS and group have the opposite impact on outcome history and neg PANAS differs between the groups).

Low and high MDQ groups combined	x	y	z	max z-score	p-value (2-tailed)	# voxels
**Reward utility (chosen - unchosen) at choice**						
Activation						
Precuneus, primary motor area (M1), caudal cingulate zone (CCZ), supplementary motor area (SMA), posterior rostral cingulate zone (RCZp) [2]	-12	-44	50	4.22	2.08E-13	3106
Area 9/46 and 45a and 47m and 47o and IFS [2,3,4]	48	8	44	4.85	6.16E-13	2961
Temporal lobve (right)	60	-28	-2	4.47	4.86E-11	2401
Temporoparietal junction (TPJa) [1] (left)	-48	-44	18	3.93	2.90E-10	2184
Spanning precuneus and intracalcarine cortex	-18	-62	12	3.94	4.20E-09	1867
Area 47m and 47o (left) [2]	-40	42	-4	4.02	1.19E-07	1472
Superior parietal lobe (SPLA) [1] (left)	-32	-40	54	3.82	1.58E-04	812
Area 8a [3] (left)	-46	4	42	4.03	4.28E-04	728
Area 8m [2]	4	44	38	5.11	8.64E-03	493
**Loss utility (chosen - unchosen) at choice**						
Activation						
Rostral cingulate zone (RCZa) [2]	12	28	26	3.26	2.06E-02	431
**Last trial's win/loss magnitude (signed) at choice**						
Activation						
Ventral striatum (bilateral) and ventromedial prefrontal cortex (14m and 11m) and medial frontal pole (FPm) [2]	-18	12	-8	4.37	4.52E-13	3084
Occipital cortex	-16	-84	-2	3.7	1.72E-02	454
**Win/loss magnitude (signed) at outcome**						
Activation						
Ventral striatum and vmPFC (14m) [2]	14	12	-8	8.88	1.88E-30	9977
Inferior parietal lobe (IPLA) [1], left	-58	-18	26	4.65	2.80E-08	1708
Area 8m [2], left	-16	38	42	5	3.58E-07	1442
Primary motor area (M1) [2], right	20	-28	72	4.06	4.76E-07	1409
Occipital lobe, left	30	-88	-6	4.94	3.10E-06	1208
Precuneus, bilateral	-16	-52	12	4.58	9.78E-06	1093
Occipital lobe, right	-28	-92	4	4.72	2.56E-04	792
Temporal lobe, left	-56	-38	-12	4.09	3.22E-04	772
Inferior parietal lobe (IPLE, IPLD, IPLC) [1], left	-42	-66	40	5.47	8.48E-04	690
Cerebellum (right)	42	-70	-38	3.57	3.32E-02	408
Deactivation						
Pre supplementary motor area (pre-SMA) [2]	2	16	52	4.72	0.00033	770

Table S9. General task brain (de)activations (related to Figure 3). Data across the low and high mood instability (MDQ) groups was combined to identify general brain (de)activations during the task. Coordinates are reported in MNI space. Significance was determined using cluster-based thresholding (methods section "FMRI analysis – whole-brain"), with inclusion threshold: z=2.3 and significance p<0.05 two-tailed. The maximum z-value of the cluster, the p-value and number of voxels are given for each cluster. Anatomical labels are based on: [1] (2)) [2] (3), [3] (4), [4] (5), [5] (6).

<u>**A Low vs high MDQ groups**</u>	x	y	z	max z-score	p-value (2-tailed)	# voxels
**Last trial's win/loss magnitude (signed) at choice**						
Low > high MDQ						
Medial frontal pole (FPm), area 9m [2]	-10	56	16	3.52	0.0378	398
<u>**B Bipolar lithium vs. placebo groups - exploratory**</u>						
**Win/loss magnitude (signed) at outcome**						
Placebo > Lithium						
Dorsolateral preforntal cortex (Area 46 [5]) and lateral frontal pole [2], Inferior frontal sulcus (IFS), (right)	38	48	0	3.49	0.00898	503

Table S10. Whole-brain group comparisons (related to Figure 4). A) Comparisons of the low vs high mood elevation volunteers. Repeating the group comparisons in the ROI, but excluding participants from the high MDQ group who had a BD diagnosis (n=5), results were still significant: estimate = 1.00, 95%CI=[0.6; 1.39]. B) Comparisons of the patients with BD assigned to placebo or lithium. All cluster-based thresholded, inclusion threshold: z=2.3, significance p<0.05 two-tailed. The maximum z-value of the cluster, the p-value and number of voxels are given for each cluster. Anatomical labels are based on: [1] (2)) [2] (3), [3] (4), [4] (5), [5] (6).

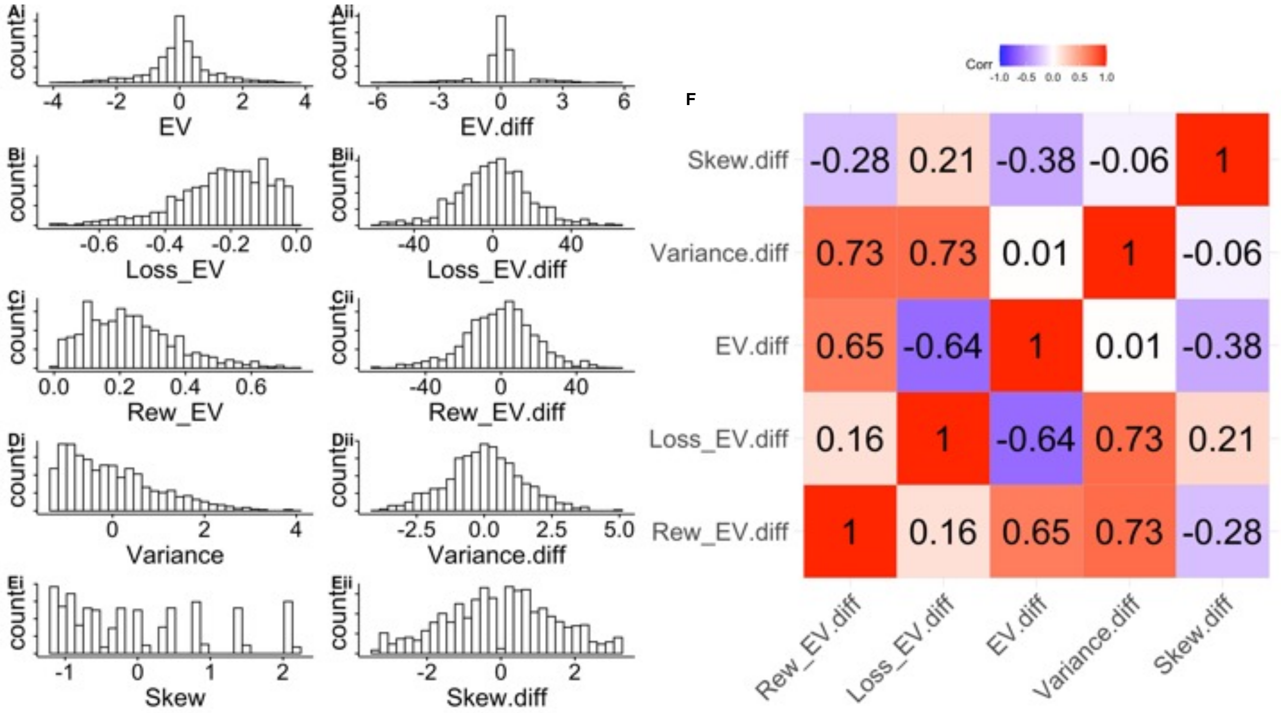

Figure S1. Illustration of task schedule (related to Figure 1). A)-E) Distributions for total expected value or ‘utility’ (EV, i.e. for each option: probability*reward magnitude + (1-probability)*loss magnitude, assuming loss magnitude is coded as negative number), loss expected value ((1-probability)*loss magnitude), reward expected value (probability*reward magnitude), variance and skew across all trials of the experiment (see supplementary methods [2B]). i) shows the distribution of these values across all options and ii) the distribution of left minus right (‘diff’) options. F) Correlation between the task properties. Of note, as expected, variance and total expected value are highly correlated with both reward and loss expected value differences.

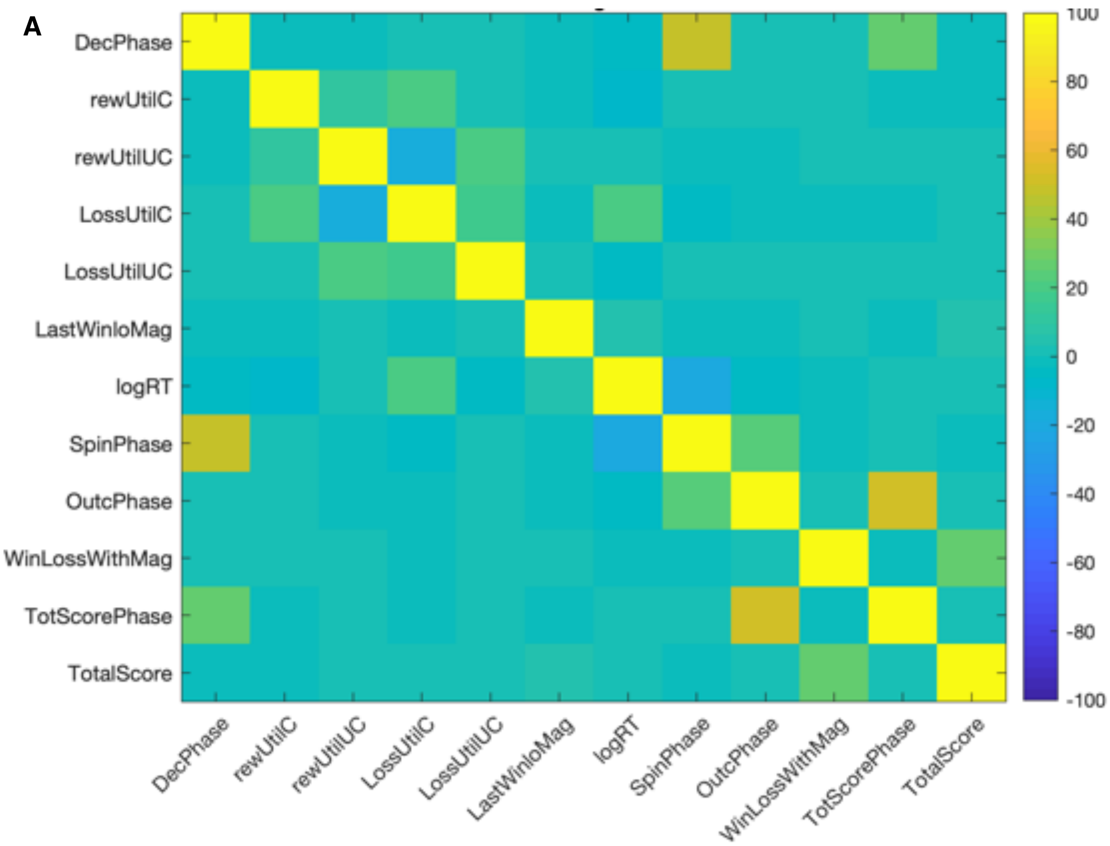

Figure S2. FMRI designs. Correlations of the haemodynamically convolved regressors for FMRI design 1 (A) and design 2 (B). No value regressors exceeded correlations of r>0.5 with any other regressors or confounds. Abbreviations: chosen reward utility (rewUtilC), unchosen reward utility (rewUtilUC), chosen loss utility (lossUtilC), last trial's outcome, i.e. points won or lost, e.g. +10 or -20 (LastWinLoMag), current trial's outcome (WinLossWithMag), relative reward utility (rewUtilCmUC), interaction between last trial's outcome and the current trial's loss utility (LastWinLoMagxLossUtilCmUC).

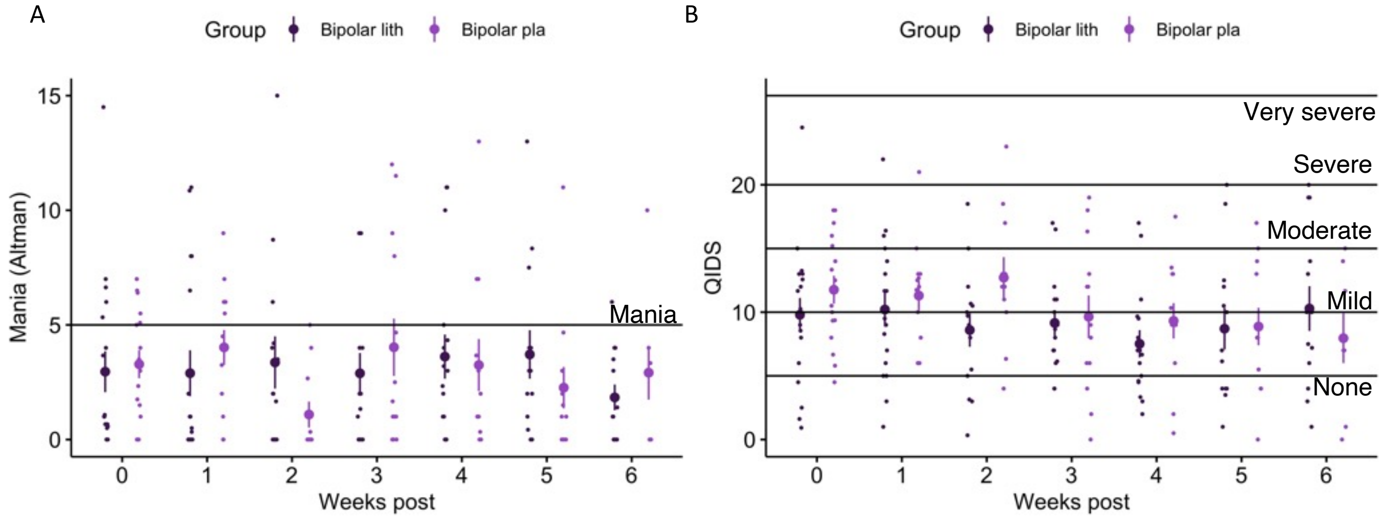

Figure S3. Mania (Altman Self Rating Mania Scale, A) and depression (Quick Inventory of Depressive Symptomatology, B) over the course of the study in patients with BD. Week zero is the average value of all weeks pre randomization (i.e. baseline) to lithium (black) vs. placebo. (purple) Horizontal lines show standard cut-offs. There were no significant differences between the groups (result of a regression predicting Altman or QIDS based on group, time and group*time, controlling for age and gender: group*time interaction: Altman 0.018, 95% CI [-0.10; 0.14]; QIDS: 0.05, 95% CI: [-0.1; 0.2]; main effect of group: Altman -0.70, 95% CI [-3.5; 2.0]; QIDS: -0.09, 95% CI: [-0.10; 0.02]; main effect of time: Altman: -0.01, 95% CI [-0.10; 0.07]; QIDS: -0.09, 95% CI: [-0.19; 0.02]).

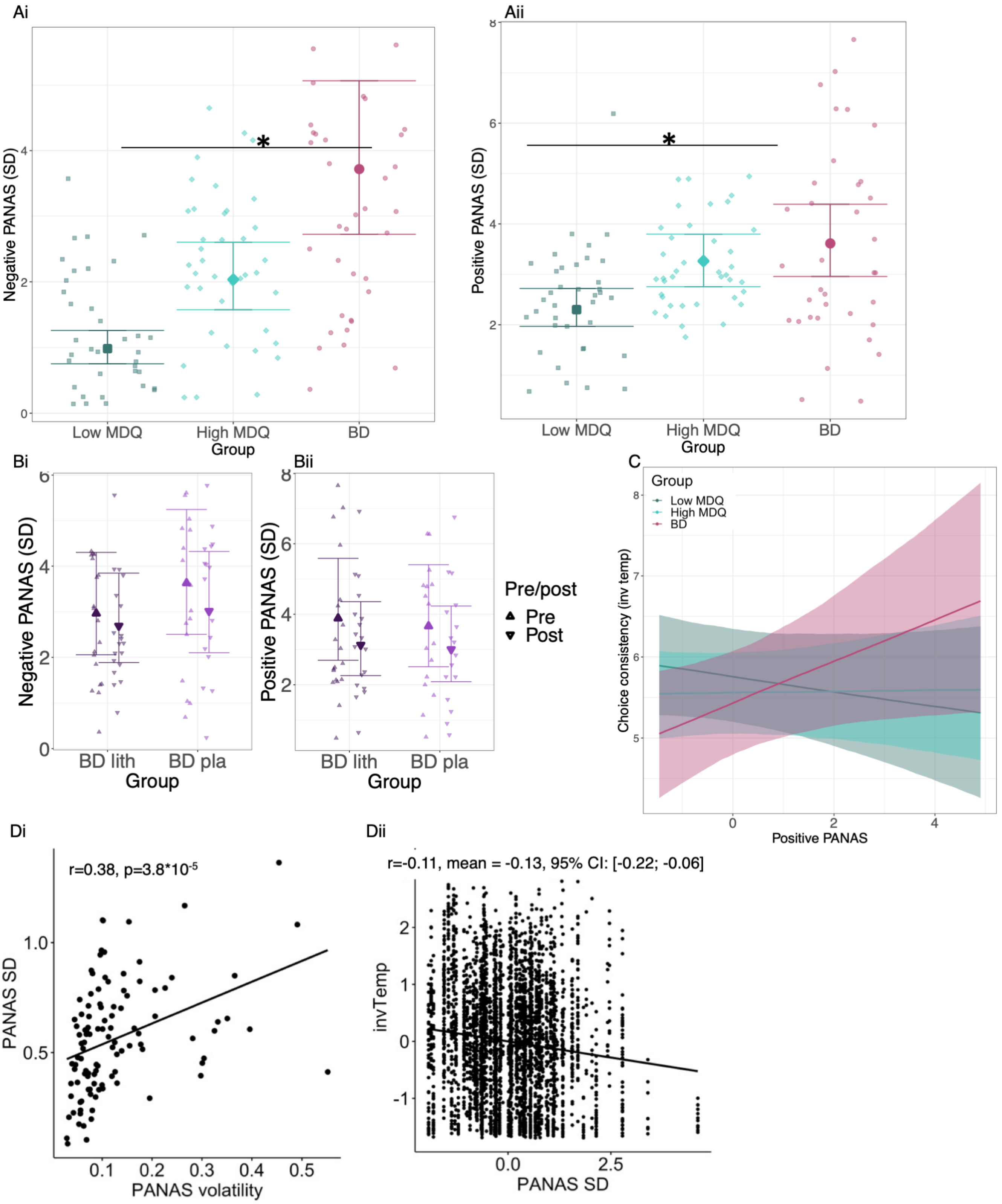

Figure S4. Mood (PANAS) variability (standard deviation). A and B as previously reported in Panchal et al. (7), shown here for ease of accessibility together with results relating mood to behaviour. A) BD gradient (ordered factor with low MDQ < high MDQ < patients with BD pre assignment to placebo or lithium) is linked to increased variability (standard deviation) for both positive and negative mood (PANAS). B) In contrast, lithium (as interaction term drug (lithium/placebo) * time (pre/post) does not affect variability of mood. See Table S8 for statistical values. C) Linking daily ratings of positive PANAS to inverse temperature (choice consistency) revealed an interaction with group (see Table S8). D) We adapted a more comprehensive model of mood variation from Pulcu et al. (1) (see supplementary methods [4] Bayesian mood instability models), fitting both mood variability and the links of mood variability to behaviour. Di) In the model, we captured separately the noisiness of mood ratings (PANAS SD, standard deviation) and the change in the average

underlying moods (PANAS volatility). The groups differed in both measures of mood instability. Group was not included in the models. To test how predictive mood instability was for group, we trained regression models for out-of-sample leave-one-out predictions (see supplementary methods [4]). We found that for a model trained to predict low vs high MDQ group, % correct classification prediction was 72% (chance: 46%). When training to predict the three groups, classification was 61% correct (either when trained on all participants or omitting those that had a diagnosis of BD in the high MDQ group; chance: 32% correct). For the high MDQ participants with a BD diagnoses, 0% were misclassified as BD. Dii) The higher the mood variability (PANAS SD), the less consistent participants' choices (i.e. lower inverse Temperature), mean =-0.07, 95%CI [-0.15; -0.002]. We illustrate here the data across all measurements, but statistics were done in the full model taking the hierarchical structure of the data (i.e. several days per participant) into account. For other parameters, no consistent results emerged (i.e. changing the modelling approach slightly to capture mood instability and its link to behaviour in separate models meant that some results disappeared that were significant in the full model, suggesting that they were at least less reliable).

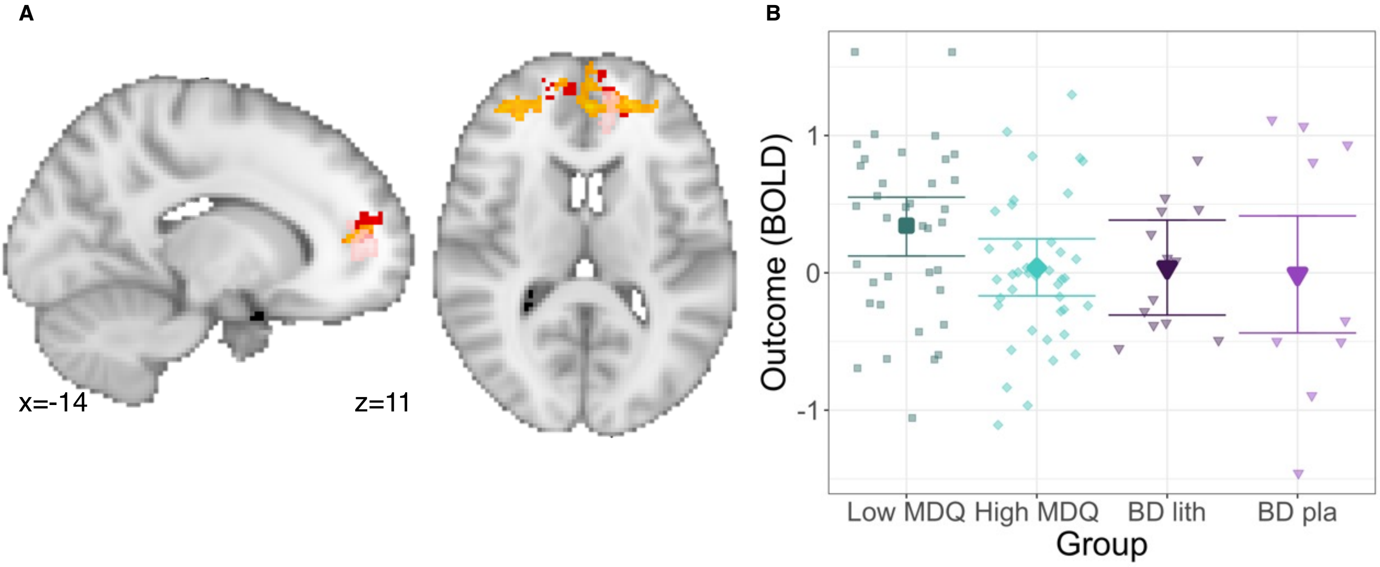

Figure S5. Whole-brain individual difference analyses. A) To complement Figure 4Aiii, we repeated the analyses whole brain, linking individual differences in the behaviour (at home) for the outcome history effect to neural signals for the last trial's outcome at choice (orange) and for the reward/loss outcome signal (pink). Significant ($p<0.05$) whole-brain clusters overlapped with the area of the group difference in the last trial's outcome signal at choice between the high and low MDQ groups (red, see Figure 4Ai). In particular, the result for the current trial outcome is noteworthy as that activation did not show any group differences. Together, this highlights a role for this medial frontal polar area in the outcome history effects. B) Despite the absence of whole-brain corrected differences between the high and low MDQ group for the outcome reward/loss activations, we also tested for group differences in the region of group differences for the last trial's outcome at the time of the choice (Figure 4A, red activation in A). We found that in fact, the low MDQ group shows a greater outcome-related activation than the high MDQ group (mean = 0.5, 95% Bayesian CI: [0.06; 0.93]). There were no differences between the BD lithium and BD placebo groups (mean=-0.1, 95% CI: [-0.92; 0.76]). Participant numbers: n=99 (all available participants across the four groups).

Supplementary methods

[1] Additional participant information

[1A] Exclusion criteria

Healthy volunteers with low and high mood instability. Common inclusion criteria across both groups were: over 18 years old, no current medication (other than contraceptive pill), no use of antidepressants, antipsychotics, lithium or anticonvulsant medication in the last 6 weeks, no contraindication to MRI or MEG. Participants were recruited based on their scores of mood instability (MDQ). The questionnaire includes first questions describing symptoms of bipolar and second a question about whether the different symptoms occurred at the same time. Participants were included in the low MDQ group if their number of reported symptoms was 5 or less. Participants were included in the high MDQ group if they reported 7 or more symptoms and report that they have happened at the same time. Additionally, in the low mood instability group, we excluded participants with a current or past diagnosis of an axis 1 psychiatric disorder (assessed using DSM-IV interview) or a first degree relative with bipolar disorder. In the high mood instability group, we excluded participants with a current or past diagnosis of an axis 1 psychiatric disorder other than bipolar disorder I or II, major depression or anxiety disorders.

Participants diagnosed with bipolar disorder. Inclusion criteria: over 18 years old, meeting criteria for BDI, BDII or BDNOS as assessed using the Structured Clinical Interview for DSM-V Axis I Disorders (SCID-I), clinically significant mood instability (established through interview), not currently suicidal (currently suicidal assessed as a score of ≥ 4 on the C-SSRS (Columbia Suicide Severity Rating Scale Score (8)), no counterindications to lithium (assessed through pre-treatment tests including renal, cardiac, thyroid and parathyroid functions), not currently taking any psychotropic drugs that could not be withdrawn, not requiring acute treatment so that placebo would be inappropriate, participation in a previous research trial in the past 12 weeks. Participants with counterindications to MRI scanner were included in the behavioural part of the study.

All participants. When inspecting the PANAS daily mood ratings, we noticed that for some participants on some days, every single response (to all positive and negative items was zero). This suggests a technical problem. We set the data from these days to zero. There were 25 participants (across all groups) for who this happened for more than 10% of measurements. Because of using hierarchical models, we could include most participants for all analyses. Specifically, we could include all participants that did not have a standard deviation of zero for the mood measures (this excluded 1 participant for the analyses for negative PANAS , 0 for positive PANAS and 2 for the analyses of changes in mood before and after the task).

[1B] Larger study – full information

Volunteers:

The data presented here was part of larger study (CONBRIO, Collaborative Oxford Network for Bipolar Research to Improve Outcomes: The cognitive neuroscience of mood instability; Cognition and Mood Evolution across Time (COMET) – MSD-IDREC-C2-2014-023). Participants who expressed interested in the study were given an electronic version of the information sheet and the Mood Disorder Questionnaire (MDQ). If they scored in either the 'low MDQ' or the 'high MDQ' category they were invited for a first study visit.

During the first study visit, inclusion and exclusion criteria were checked. Participants were also screened for psychiatric disorders using the Structured Clinical Interview for DSM Disorders (SCID) (9). Participants completed several questionnaires, including Barratt Impulsiveness Scale (10), Sleep Condition Indicator (11), Maclean Screening Instrument (12), Affective Lability Scale (13), Affect Intensity Measure (14). Participants were set up with and instructed how to use devices to measure their activity (GeneActiv watch and FitBit) over the ten weeks study period. They were given an iPad mini and trained on four cognitive tasks: 'wheel of fortune' – risky decision making (presented here), 'guess the gap' – performance learning, 'fractals' – stimulus-outcome learning, 'whack-a-t' – implicit spatial learning.

Over a period of ten weeks, they were asked to complete these tasks five times a week and to wear the GeneActiv and FitBit devices as much as possible. They were also asked to complete clinical questionnaires (Quick Inventory of Depressive Symptomatology (15) , Altman self-rating mania scale (16), Generalized Anxiety Disorder-7 (17), EuroQol-5 [health-related quality of life] (18)) on the True Colours mood monitoring system (19) once a week. They were also asked to stay within the recommended daily alcohol intake levels throughout.

In the beginning of the ten-week period (weeks one or two) and in the end (weeks nine and ten), participants attended an MRI scan and a MEG scan. Due to MEG scanner downtime, only 24 participants received the MEG scan. During both MRI scans, resting state and structural data was obtained. At the first scan, additional the 'wheel of fortune' was measured. At the second MRI scan, 'fractals' and 'guess the gap' was measured, as well as diffusion tension imaging and fluid-attenuated inversion recovery.

Participants diagnosed with bipolar disorder:
Participants first took part in a screening visit in which inclusion criteria were checked and informed consent was taken. Using the SCID-I, a diagnosis check was done. In addition, demographic and clinical information was obtained, including duration of illness, previous use of psychotropic medicines, family history of mood disorders, presence of comorbid borderline personality disorder and attention deficit hyperactivity disorder and current suicidal ideation, concomitant medication and substance use and a physical examination. If blood samples have not been taken as part of routine monitoring, they were taken at this visit. Two sets of samples were taken, one was sent to the pathology lab for analysis and the other was retained as replacement for samples lost/damaged in transit and for storage for future research. Tests included urea and electrolytes, full blood count, fasting blood glucose, glycosylated haemoglobin (HbA1c), blood lipid profile, LFTs, T4, T3, TSH, thyroid antibodies, PTH, vitamin D, eGFR, Cystatin C and NGAL and inflammatory markers CRP and IL-6. A sample was taken to measure calcium level using the InSight™ Electrolyte Analyser located in the NIHR-CRF. Weight/BMI, pulse and blood pressure were also recorded, and an ECG was performed. Participants were given an iPad mini and trained on the same cognitive tasks as the healthy volunteers described above. They were also set up with the True Colours system to rate weekly mood and on Mood Zoom (20) to rate daily mood. Participants were also given activity monitors. They were also given saliva swabs.

Before being randomised to lithium or placebo, all participants completed two weeks of daily cognitive tasks, mood and activity measurements at home (though some participants completed up to 30 days due to logistic challenges). Then, they were randomised and performed six weeks of cognitive tasks, mood and activity measurements.

In the beginning of the six weeks period (week one or two), they completed an MRI and a MEG scan using the same scans as described for the healthy volunteers.

Randomisation: The first 10 participants were fully randomly assigned to avoid predictability, while for subsequent participants, an algorithm was used to minimize differences in age (<25 or >25 years) and gender

between the two groups. In the lithium group, participants were titrated to doses producing plasma levels of 0.6-1 mmol/L (see supplementary methods 1C for dosing details).

[1C] Lithium dosing information

In the lithium group, participants were prescribed an initial dose of 400g/day, unless there was a clinical indication to start at a lower dose. During this phase, participants attended brief assessments at 4-days, 8-days and between 2 and 3 weeks post-randomisation to review lithium levels by a psychiatrist (if lithium level ≤ 0.3 mmol/L, dose increased to 800mg/day; if lithium level between 0.4 to 0.5 mmol/L, dose increased to 600mg/day; if lithium level 0.6 -1.0mmol/L, continued current dose; if lithium level ≥ 1.0mmol/L, decrease dose by 200mg/day or 400mg/day as found appropriate by psychiatrist), receive additional supplies of lithium/ placebo as needed and were asked about adverse events. Participants took part in one neuroimaging session in week 3 or 4. During the trial, participants were asked to complete the cognitive tasks daily.

[2] Computational modelling, additional information

[2A] Decision making model validation

We validated our computational models using simulations (21,22). We simulated 400 participants with parameter values (mean and standard deviations) drawn from a uniform distribution in the 95% range of the parameters for real individual participants. For each participant, we simulated 47-50 sessions (uniform distribution). Parameters for single sessions were drawn from normal distributions of simulated participants' means, standard deviations and linear effects of days. Simulated data was then fitted using same approaches as above. To speed up fitting of data, variational Bayesian approximation (23) was used unless control indices (pareto smoothed importance sampling, khat >0.7 (24)) suggested unsuccessful fitting even after increasing number of samples and decreasing tolerance, in which case sampling was used. When fitting the models, initially, 4 chains, with each 15,000 iterations were drawn and the target acceptance rate (adapt_delta, (25)) was set to 0.85. Whether models had been fit appropriately was checked using a criterion of R-hat (measure of mixing of chains) < 1.1 and absence of divergent samples. If these were not fulfilled, number of iterations were increased by 50% and adapt_delta was increased towards 1 (by 50% of distance from 1). This was repeated until all models converged.

To validate the model, we then checked the correlations between true and fitted values for mean and standard deviation of parameters across individual subjects (table S1).

[2B] Alternative decision-making models and model comparison

In addition to the models in the main text (section Computational models – decision making), we also considered two other classes of models (see table S2 for full list). First models that incorporated probability and magnitude distortions according to prospect theory (26) (class 'M2'):

$$Utility_{left} = Prob\_adj * Mag_{rew}^{\delta r} - \lambda * (1 - Prob\_adj) * Mag_{loss}^{\gamma l}$$

Where

$$Prob_{adj} = \frac{Prob^{\varepsilon}}{(Prob^{\varepsilon} + (1 - Prob)^{\varepsilon})^{\frac{1}{\varepsilon}}}$$

Here, ε is the probability distortion; δr is the distortion of the reward magnitudes; δl is the distortion of the loss magnitudes and λ is the weighting of the loss (scaled mag * scaled prob).

We also fitted further versions of this model, leaving out the probability distortion, the loss scale or the magnitude distortions.

Of note, due to there only being 20 trials available per session, not all of these models could be fitted. Specifically models that contained probability distortion could not be fitted and models with a loss weight in addition to exponential scaling of loss could not be fitted.

In these models, the outcome history effect was initially included in the exponential of the loss magnitude:

$$Mag_{loss}^{(\gamma l+\gamma * PrevOutc_{\frac{win}{loss}})}$$

However, we noted that, potentially due to the difficulty of estimating parameters that are used as an exponent, parameter recovery for the outcome history effect in this model was not very good. Therefore, we also included the outcome history parameter as a linear weight of the exponentially distorted magnitudes.

The second set of models allowed participants to differ in their relative weighting of expected value, variance and skew (27) (class ‘M3’):

$$Utility_{left} = ExpectedValue + \alpha * Variance + \chi * Skew$$

Where:

$$ExpectedValue\ (EV) = Prob * Mag_{rew} - (1 - Prob) * Mag_{loss}$$

(given that Mag_{loss} is a positive number).

$$Variance = Prob * (Mag_{rew} - EV)^2 + (1 - Prob) * (-Mag_{loss} - EV)^2$$

$$Skew = \frac{Prob * (Mag_{rew} - EV)^3 + (1 - Prob) * (-Mag_{loss} - EV)^3}{Var_{2}^{3}}$$

Again, we fitted this model also without the weighing for skew or without the weighing for variance.
In this model, outcome history was captured as impacting the weighing of variance or skew or both.

To compare models, we used the Akaike Information Criterion (AIC) (28), which combines the log likelihood with the number of model parameters to avoid selection of over-parameterized models. Models were fit across all sessions from each participant, including for each parameter (other than outcome history effects, see table S5) a linear effect of day:
$Parameter_t$ = $Parameter_{t0}$ + day_effect*current_day
AIC values were summed across participants. Participants for whom not all models could be fit were omitted (n=2).

[2C] Bayesian models – additional information for standard settings
Regression models were computed with the BRMS toolbox (29) which uses the Bayesian programming language Stan (30). The key advantages of the Bayesian approach are: priors can be defined to ease fitting, particularly when little data is available (as here only 20 trials per session); models can be hierarchical and account for individual differences in each parameter (i.e. taking into account data consistent of within and between subject measurements, e.g. several data points per person and several subjects); variability in measurements across people can be taken into account.

Linear non-hierarchical regression
All regression estimates (parameters) were given flat priors for all parameters and 5000 iterations for each of four chains were drawn (target acceptance rate, adapt_delta = 0.9). Model fit was checked using criterion of Rhat <1.1 and the absence of divergent samples (25). If models did not converge, iterations and adapt_delta were increased step-wise, up to a max of 25312 iterations and adapt_delta = 0.991. If fitting was then still not successful (only the case for Prospect Theory models, class M2, listed in [2B] above), the sessions were left out from model comparisons.

Linear hierarchical regressions

To ease fitting (31), all regression estimates were given weakly informative priors, normal(0,5). 6,000 iterations for each of 4 chains were drawn (target acceptance rate, adapt_delta = 0.9). Model fit was checked as above for non-hierarchical models. Significance follows the standard definition of the Bayesian 95% Credible Interval not including zero. To compare individual groups, the same model was fitted with group as an unordered factor and posthoc tests were then done using the emmeans package (32) (again using 95% Credible Intervals to define significance). Results of regressions are illustrated as conditional effects, i.e. all other variables are set to their mean. We computed mean parameters for individual participants to relate to neural activity.

Computational decision-making models – prior settings

For each parameter weakly informative priors were specified for models for the longitudinal or the FMRI data for each session (longitudinal) or person (FMRI): inverse temperature (β): cauchy(5,3), weighting of the loss utility (λ): cauchy(-1,1), the impact of the previous trial's win/loss on the weighting of the loss utility (γ); group level standard deviations: inverse temperature (β): cauchy(0,3), all other parameters: cauchy(0, 1).

When fitting the decision-making models, number of iterations and adapt_delta were increased until fit indices suggested appropriate fit, as described above.

FMRI

Computational models were fitted as for the longitudinal data, i.e. first separately for each individual participant before then comparing the computational model parameters across groups using non-hierarchical models (as one FMRI session per person).

[2D] Regressions relating mood, task outcomes and behaviour

We used hierarchical regression models to test for group differences in the impact of task outcomes on mood:
Mean: Happiness (post minus pre) ~ 1 + Task outcomes* group + Task outcomes + group + day + Age + Gender + (1 + day + outcome | ID)
And error term: sigma ~ 1 + group + age + gender + (1|ID)
Where outcome was either the total wins in the daily task, the total losses or the total wins minus losses. Group was coded as monotonic factor.

In addition to the happiness VAS that was measured before and after the task, we also measured mood using a more detailed questionnaire (PANAS-SF) before the task. We used this to replicate previous findings (1,20,33,34) of mood instability related to bipolar disorder (with hierarchical models):
Mean: PANAS ~ 1 + day + group + age + gender + (1 + day |ID)
Error term: Sigma ~ 1 + group + age + gender + (1|ID)
Where PANAS was either the positive or the negative PANAS score.

[3] MRI scan

[3A] MRI acquisition sequences

Scan protocols were similar across both sites and differences are highlighted. T1-weighted structural images were acquired with the settings TR=3 sec, TE=4.71 msec (4.65ms for second site [some bipolar patients]), TI (inversion time) = 1.1 sec, 1x1x1 mm voxel size, 256x176x224 mm grid, flip angle = 8°, phase-encoding direction = R-L, GRAPPA (Generalized autocalibrating partially parallel acquisition) = 2. Functional images were acquired using a Deichmann echo-planar imaging (EPI) sequence with TR=3 s, TE=30 ms, 3x3x3 mm voxel size, 87° flip angle, 30° slice angle and z-shimming to reduce signal distortions as well as dropout in

medial orbitofrontal areas (35). A fieldmap with dual echo-time images (TE1 = 5.19 ms, TE2 = 7.65 ms, whole brain coverage, voxel size 3.5 × 3.5 × 3.5 mm) was obtained for each subject to allow for corrections in geometric distortions induced in the functional images.

[3B] FMRI preprocessing

We used FSL (36) version 6.00 for standard image preprocessing and analysis (suppl. Methods [4B]. We used FSL's BET (37) on the high-resolution structural MRI images and fieldmaps images to separate brain matter from nonbrain matter. We used the structural images to register functional images in MNI space using nonlinear registration as implemented in FNIRT (38). Functional images were corrected for motion using FSL's MCFLIRT (39), corrected for geometric distortions using FSL's FUGUE (FMRIB's Utility for Geometrically Unwarping EPIs) and spatially smoothed with a Gaussian kernel of 5mm full-width half-maximum. Finally, images were then high-pass filtered with a 3 dB cutoff of 100s.

[3C] FMRI analysis

Data were pre-whitened before analysis (40). The fMRI design was as follows (see Figure S2 for design correlation matrix): We included four boxcar regressors capturing the different phases of each trial: the decision phase (aligned to the onset of the decision phase and lasting until participants could make a choice), the spinning phase (aligned to when the indicator on the chosen wheel of fortune started moving and lasting until it stopped), the outcome phase (the time the outcome was shown to participants and lasting until it disappeared from the screen) and the total score phase (aligned to when the screen with the total score was shown and lasting until it disappeared). Here the decision and the outcome phase are the main phases of interest, the others are included as control regressors. We also included parametric boxcar regressors aligned to same onsets as the phases described above, but with duration one second. All regressors were z-score normalized within each participant. In the decision phase, we included separate regressors for the reward and loss utilities (i.e. probability x magnitude) of the chosen minus the unchosen options, a regressor for last trial's outcome (win loss, including the magnitude therefore, e.g. +10 or -20), as well as participants' log-transformed reaction time as a control regressor. In the outcome phase, we included a regressor indicating the current trial's outcome (win/loss, including the magnitude thereof). As control regressor we also included the total score phase with total scores as parametric value. All regressors were convolved with a double-gamma hemodynamic response function.

[4] Bayesian mood instability models

Pulcu et al. (1) proposed a model of mood variations that captures simultaneously variability in mood ratings and drifts (volatility) in the mean mood ratings. We adapted this model here (simplifying due to less data being available than in Pulcu et al., that neither volatility nor standard deviations changed over time and fit to both positive and negative PANAS simultaneously). The same model also captured relationships between PANAS standard deviation and behaviour. The key equations of the model included:

$PANAS_{ratings[t]} \sim normal(PANAS_t, PANAS_{sd})$

$PANAS_t \sim normal(PANAS_{t-1}, PANAS_{volatility})$

Where volatility and standard deviation of PANAS ratings were shared across positive and negative PANAS. In contrast, PANAS values ($PANAS_t$ above) were captured separately for positive and negative PANAS.

$Behaviour \sim normal(b_0 + b_{PANAS\_sd} * PANAS_{sd} + b_{testing\ day} * testing\_day, behaviour_{sd})$

The model was fit to data from all participants who had at least 5 data points available for all measurements and standard deviations of both positive and negative PANAS above 0 (i.e. who did not always report exactly the same mood). Models were fitted as hierarchical models (mixed effects models), with group level parameters (mean and standard deviations) fitted for $PANAS_{sd}$, $PANAS_{volatility}$, $b_{testing\_day}$ and $behaviour_{sd}$. Parameters for individual participants were then drawn from the thus defined normal distributions. $PANAS_t$ for positive and negative PANAS was fitted as one parameter per person per day (with the temporal order constraints as described in the regressions above). All parameters were given priors normal(0,1), for constraint parameters (i.e. standard deviations and volatility), log transformations were used.

To test how predictive mood instability was of group membership, we first fitted a simpler model of only the PANAS scores, excluding the behaviour, separately for each person. We then used the $PANAS_{sd}$ and $PANAS_{volatility}$ to predict group membership in a leave-one-out cross-validation procedure, each time fitting a model of the form:

Group ~ 1+ $PANAS_{sd}$ + $PANAS_{volatility}$

The model was fit to training data (i.e. all participants apart from one) and used to predict the test data (the left out participant). We trained models separately predicting low vs. high MDQ and predicting all three groups.

References

1. Pulcu E, Saunders KEA, Harmer CJ, Harrison PJ, Goodwin GM, Geddes JR, Browning M (2022): Using a generative model of affect to characterize affective variability and its response to treatment in bipolar disorder. *Proceedings of the National Academy of Sciences* 119: e2202983119.
2. Mars RB, Jbabdi S, Sallet J, O'Reilly JX, Croxson PL, Olivier E, *et al.* (2011): Diffusion-weighted imaging tractography-based parcellation of the human parietal cortex and comparison with human and macaque resting-state functional connectivity. *Journal of Neuroscience* 31: 4087–4100.
3. Neubert F-X, Mars RB, Sallet J, Rushworth MFS (2015): Connectivity reveals relationship of brain areas for reward-guided learning and decision making in human and monkey frontal cortex. *PNAS* 112: E2695–E2704.
4. Sallet J, Mars RB, Noonan MP, Neubert F-X, Jbabdi S, O'Reilly JX, *et al.* (2013): The Organization of Dorsal Frontal Cortex in Humans and Macaques. *J Neurosci* 33: 12255–12274.
5. Diedrichsen J, Balsters JH, Flavell J, Cussans E, Ramnani N (2009): A probabilistic MR atlas of the human cerebellum. *NeuroImage* 46: 39–46.

6. Neubert F-X, Mars RB, Thomas AG, Sallet J, Rushworth MF (2014): Comparison of human ventral frontal cortex areas for cognitive control and language with areas in monkey frontal cortex. *Neuron* 81: 700–713.

7. Panchal, P, Nelissen, N, McGowen, N, Atkinson, LZ, Saunders, KEA, Harrison, PJ, *et al.* (in submission): Identifying mood instability and circadian rest-activity patterns using digital remote monitoring and actigraphy in participants at risk for bipolar disorder.

8. Nilsson ME, Suryawanshi S, Gassmann-Mayer C, Dubrava S, McSorley P, Jiang K (2013): Columbia–suicide severity rating scale scoring and data analysis guide. *CSSRS Scoring Version* 2: 1–13.

9. First MB (2014): Structured clinical interview for the DSM (SCID). *The encyclopedia of clinical psychology* 1–6.

10. Patton JH, Stanford MS, Barratt ES (1995): Factor structure of the Barratt impulsiveness scale. *Journal of clinical psychology* 51: 768–774.

11. Espie CA, Kyle SD, Hames P, Gardani M, Fleming L, Cape J (2014): The Sleep Condition Indicator: a clinical screening tool to evaluate insomnia disorder. *BMJ open* 4: e004183.

12. Zanarini MC, Vujanovic AA, Parachini EA, Boulanger JL, Frankenburg FR, Hennen J (2003): A screening measure for BPD: The McLean screening instrument for borderline personality disorder (MSI-BPD). *Journal of personality disorders* 17: 568–573.

13. Oliver MN, Simons JS (2004): The affective lability scales: Development of a short-form measure. *Personality and individual differences* 37: 1279–1288.

14. LARSEN RJ (1984): *Theory and Measurement of Affect Intensity as an Individual Difference Characteristic (Temperament, Emotion, Arousal)*. University of Illinois at Urbana-Champaign.

15. Rush AJ, Trivedi MH, Ibrahim HM, Carmody TJ, Arnow B, Klein DN, *et al.* (2003): The 16-Item Quick Inventory of Depressive Symptomatology (QIDS), clinician rating (QIDS-C), and self-report (QIDS-SR): a psychometric evaluation in patients with chronic major depression. *Biological psychiatry* 54: 573–583.

16. Altman EG, Hedeker D, Peterson JL, Davis JM (1997): The Altman self-rating mania scale. *Biological psychiatry* 42: 948–955.

17. Spitzer RL, Kroenke K, Williams JB, Löwe B (2006): A brief measure for assessing generalized anxiety disorder: the GAD-7. *Archives of internal medicine* 166: 1092–1097.

18. Group TE (1990): EuroQol-a new facility for the measurement of health-related quality of life. *Health policy* 16: 199–208.

19. Goodday SM, Atkinson L, Goodwin G, Saunders K, South M, Mackay C, *et al.* (2020): The True Colours Remote Symptom Monitoring System: A Decade of Evolution. *Journal of Medical Internet Research* 22: e15188.

20. Tsanas A, Saunders KEA, Bilderbeck AC, Palmius N, Osipov M, Clifford GD, *et al.* (2016): Daily longitudinal self-monitoring of mood variability in bipolar disorder and borderline personality disorder. *Journal of affective disorders* 205: 225–233.

21. Kolling N, Scholl J, Chekroud A, Trier HA, Rushworth MF (2018): Prospection, perseverance, and insight in sequential behavior. *Neuron* 99: 1069–1082.

22. Scholl J, Trier HA, Rushworth MF, Kolling N (2022): The effect of apathy and compulsivity on planning and stopping in sequential decision-making. *PLoS biology* 20: e3001566.

23. Kucukelbir A, Ranganath R, Gelman A, Blei DM (2015): Automatic variational inference in Stan. *arXiv preprint arXiv:150603431*.

24. Yao Y, Vehtari A, Simpson D, Gelman A (2018): Yes, but did it work?: Evaluating variational inference. *International Conference on Machine Learning* 5581–5590.

25. Gelman A, Vehtari A, Simpson D, Margossian CC, Carpenter B, Yao Y, *et al.* (2020): Bayesian workflow. *arXiv preprint arXiv:201101808*.

26. Nilsson H, Rieskamp J, Wagenmakers E-J (2011): Hierarchical Bayesian parameter estimation for cumulative prospect theory. *Journal of Mathematical Psychology* 55: 84–93.

27. Symmonds M, Wright ND, Bach DR, Dolan RJ (2011): Deconstructing risk: Separable encoding of variance and skewness in the brain. *Neuroimage* 58: 1139–1149.

28. Vrieze SI (2012): Model selection and psychological theory: A discussion of the differences between the Akaike information criterion (AIC) and the Bayesian information criterion (BIC). *Psychological Methods* 17: 228–243.

29. Bürkner P-C (2017): brms: An R package for Bayesian multilevel models using Stan. *Journal of Statistical Software* 80: 1–28.

30. Carpenter B, Gelman A, Hoffman MD, Lee D, Goodrich B, Betancourt M, *et al.* (2017): Stan : A Probabilistic Programming Language. *Journal of Statistical Software* 76. https://doi.org/10.18637/jss.v076.i01

31. Michael Betancourt (2017): How the shape of a weakly informative prior affects inferences. Retrieved from https://mc-stan.org/users/documentation/case-studies/weakly_informative_shapes.html

32. Lenth RV (2021): *Emmeans: Estimated Marginal Means, Aka Least-Squares Means*. Retrieved from https://CRAN.R-project.org/package=emmeans

33. Bonsall MB, Wallace-Hadrill SMA, Geddes JR, Goodwin GM, Holmes EA (2012): Nonlinear time-series approaches in characterizing mood stability and mood instability in bipolar disorder. *Proceedings of the Royal Society B: Biological Sciences* 279: 916–924.

34. Holmes EA, Bonsall MB, Hales SA, Mitchell H, Renner F, Blackwell SE, *et al.* (2016): Applications of time-series analysis to mood fluctuations in bipolar disorder to promote treatment innovation: a case series [no. 1]. *Transl Psychiatry* 6: e720–e720.

35. Deichmann R, Gottfried JA, Hutton C, Turner R (2003): Optimized EPI for fMRI studies of the orbitofrontal cortex. *NeuroImage* 19: 430–441.

36. Smith SM, Jenkinson M, Woolrich MW, Beckmann CF, Behrens TE, Johansen-Berg H, *et al.* (2004): Advances in functional and structural MR image analysis and implementation as FSL. *Neuroimage* 23: S208–S219.

37. Smith SM (2002): Fast robust automated brain extraction. *Human brain mapping* 17: 143–155.

38. Andersson JL, Jenkinson M, Smith S (2007): Non-linear registration aka Spatial normalisation FMRIB Technial Report TR07JA2. *FMRIB Analysis Group of the University of Oxford*.

39. Jenkinson M, Bannister P, Brady M, Smith S (2002): Improved optimization for the robust and accurate linear registration and motion correction of brain images. *Neuroimage* 17: 825–841.

40. Woolrich MW, Ripley BD, Brady M, Smith SM (2001): Temporal autocorrelation in univariate linear modeling of FMRI data. *Neuroimage* 14: 1370–1386.
